# Advanced Monitoring of H_2_S Injection through the Coupling of Reactive Transport Models and Geophysical Responses

**DOI:** 10.1021/acs.est.3c10139

**Published:** 2024-06-10

**Authors:** Daniel A. Ciraula, Barbara I. Kleine-Marshall, Iwona M. Galeczka, Léa Lévy

**Affiliations:** † Nordic Volcanological Center, Institute of Earth Sciences, University of Iceland, Reykjavík 101, Iceland; ‡ GeoZentrum Nordbayern, Friedrich-Alexander-Universität, Schlossgarten 5, Erlangen-Nuremberg 91054, Germany; § ÍSOR-Iceland Geosurvey, UrĐarhvarf 8, Kópavogur 203, Iceland; ∥ Carbfix, HöfĐabakki 9D, Reykjavík 110, Iceland; ⊥ Engineering Geology, Lund University, Lund Box 117 SE-221 00, Sweden

**Keywords:** hydrogen sulfide, induced polarization, wireline logging, mineral storage, basalt, pyrite, geothermal wastewater

## Abstract

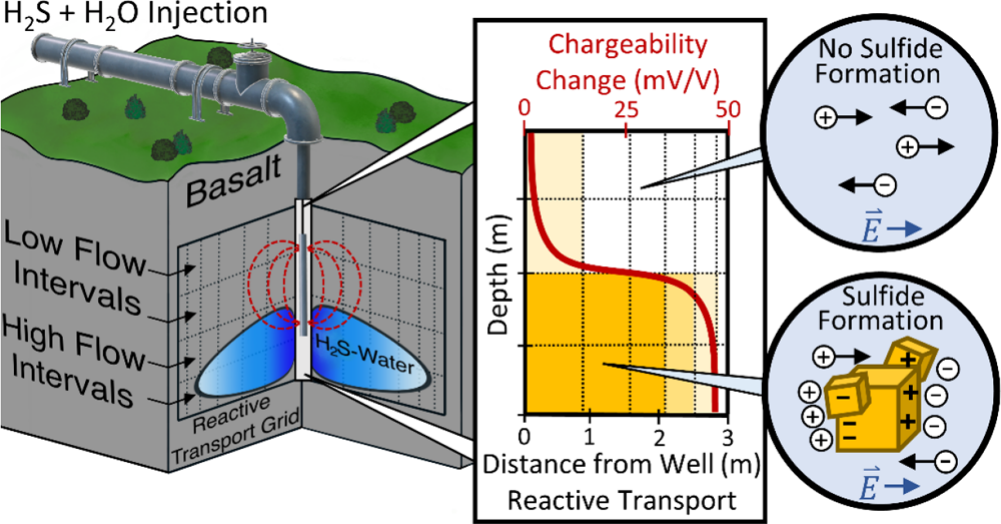

Hydrogen sulfide (H_2_S), an environmentally harmful pollutant, is a byproduct of geothermal energy production. To reduce the H_2_S emissions, H_2_S-charged water is injected into the basaltic subsurface, where it mineralizes to iron sulfides. Here, we couple geophysical induced polarization (IP) measurements in H_2_S injection wells and geochemical reactive transport models (RTM) to monitor the H_2_S storage efforts in the subsurface of Nesjavellir, one of Iceland’s most productive geothermal fields. An increase in the IP response after 40 days of injection indicates iron-sulfide formation near the injection well. Likewise, the RTM shows that iron sulfides readily form at circumneutral to alkaline pH conditions, and the iron supply from basalt dissolution limits its formation. Agreement in the trends of the magnitude and distribution of iron-sulfide formation between IP and RTM suggests that coupling the methods can improve the monitoring of H_2_S mineralization by providing insight into the parameters influencing iron-sulfide formation. In particular, accurate fluid flow parameters in RTMs are critical to validate the predictions of the spatial distribution of subsurface iron-sulfide formation over time obtained through IP observations. This work establishes a foundation for expanding H_2_S sequestration monitoring efforts and a framework for coupling geophysical and geochemical site evaluations in environmental studies.

## Abstract 2

This study interprets geochemical reactive transport models alongside geophysical induced polarization wireline logging to monitor hydrogen sulfide mineral storage in basaltic host rocks, identifying controls on its magnitude and spatial distribution at the field scale.

## 1 Introduction

Geothermal energy production offers an environmentally friendly base-load energy alternative to fossil fuels. However, as the geothermal steam contains significant H_2_S content, it contributes to anthropogenic hydrogen sulfide (H_2_S) emissions at a rate of ∼0.2 Mt/year globally and 30 kt/year in Iceland.^1−3^ The H_2_S emissions are toxic to humans, causing respiratory arrest at concentrations exceeding 530 ppm.^4^ They also pose a threat to the environment when reacting with the atmospheric oxygen to form acid rain.^5^ Recently established air quality regulations limit the H_2_S concentration to 50–150 μg/m^3^ per 24 h.^6,7^

To meet emission standards at the Nesjavellir power plant in southwest Iceland, H_2_S from the geothermal flue gas is captured, dissolved into geothermal wastewater, and injected into the basaltic subsurface.^8^ Due to high reactivity and a high divalent cations content (up to 25 wt % Ca, Mg, Fe),^9,10^ basalt has the potential to effectively mineralize H_2_S through iron sulfide (e.g., pyrite, pyrrhotite) formation upon interaction with the H_2_S-charged injection fluid.^1−3^ This mineral storage approach is similar to the CarbFix process, which has been studied extensively for the co-capture of CO_2_ and H_2_S.^3,11−17^ Although these studies suggest that H_2_S injection and its subsequent mineralization in basalt is a viable solution to store the H_2_S emissions permanently, it is crucial to establish a reservoir monitoring strategy to identify potential consequences of such injection. For example, if sulfide mineralization is sluggish, dissolved H_2_S oxidation can acidify groundwater and mobilize toxic metals from the host rock.^18−23^

The most utilized method to monitor field-scale H_2_S injections is to analyze the chemical composition of the injection reservoir fluid and its parameters.^15−17^ Such monitoring, along with geochemical numerical models and laboratory simulations, gives insights into the processes governing the dissolved H_2_S removal from the injection fluid.^1−3,11,13−16,24,25^ However, verification of the mineralization requires monitoring boreholes close to the injection wells. Furthermore, the chemical composition of water from the reservoir provides only indirect evidence of such mineralization. A potential method to acquire direct information on the magnitude and spatial distribution of the H_2_S mineralization in the storage reservoir is induced polarization (IP) wireline logging. Implementing borehole IP as a monitoring technique provides benefits of high spatial resolution and repeatability over time, enabling alteration processes to be measured along the borehole.^26,27^

The magnitude of the induced polarization response and the volume of polarized material exhibit a positive relationship.^28,29^ Previous studies have implemented the IP method to interpret mineral precipitation (i.e., sulfides and carbonates) in laboratory experiments^30−36^ and at field sites.^37−41^ Particularly for field studies, interpretation of the acquired data can be challenging due to signal noise and the presence of other IP sources that contribute to the signal, especially when dynamic processes are studied.^39,42^ To aid in the interpretation of IP measurements and to constrain physiochemical parameters, the IP surveys can be investigated alongside mechanistic models, such as geochemical reactive transport modeling (RTM).^33,39,42−44^ While laboratory studies have coupled electrochemical geophysics and RTM,^30,33^ this coupling remains unexplored for field-scale applications of mineral precipitation.

In this study, we aim to couple wireline geophysical surveying (i.e., IP geophysics) and geochemical RTM to identify the processes and physicochemical parameters (e.g., fluid and rock chemistry, degree of basalt alteration, temperature, and reservoir porosity and permeability), controlling subsurface H_2_S mineralization. The outcome of this study contributes to (1) the general understanding of engineered H_2_S mineralization, (2) enhancing the monitoring methods to improve the safety of such operations, and (3) advancing the integration of joint geochemical and geophysical interpretations.

## 2 Materials and Methods

### 2.1 Site Description

This study focuses on the geothermal power plant at the Nesjavellir high-temperature (>200 °C at <1 km depth) geothermal field (SW Iceland). Here, we provide a brief overview of the geologic setting and the H_2_S injection system at Nesjavellir, which have been previously described in detail.^11,45−47^ The lithology primarily comprises shallow hyaloclastite formations and lava flows (<400 m), with intrusions at greater depths.^47,48^ The production fluid (260–300 °C) at Nesjavellir is sourced at 1000–1500 m depth.^12^ The intensity of the subsurface basalt alteration is temperature- and depth-dependent, and it is manifested as distinct alteration zones: (1) no alteration in the upper 450 m, (2) zeolite and smectite (<200 °C), (3) mixed layer clays (200–230 °C), (4) chlorite (230–250 °C), (5) chlorite–epidote (250–280 °C), (6) epidote–actinolite (280–330 °C), and (7) amphibolite at the greatest depths (>330 °C).^46,49^

Production began at Nesjavellir in 1990, and the power plant currently outputs 120 MW_e_ of electricity and 290 MW_th_ as thermal energy for district heating. A byproduct of this energy production is geothermal wastewater, sourced from the high enthalpy production fluid. The majority of the wastewater is disposed of in shallow injection wells, including the NN-3 and NN-4 wells, which began injecting in 2004. These wells were drilled through minimally altered lavas down to 563 m (NN-3) and 422 m (NN-4) depth, and they tap the cold groundwater system at the outskirts of the geothermal site.^46,49^ Boreholes for chemical monitoring are limited at this injection site, and the closest borehole is ∼1.5 km from the injection.

Since January 29, 2021, the power station has dissolved captured H_2_S from the geothermal emissions into the wastewater (separated geothermal wastewater and condensate wastewater mixture) and has continuously injected the H_2_S-charged water into the NN-3 and NN-4 wells. Injection is under reduced conditions to prevent corrosion of the infrastructure resulting from the formation of sulfuric acid following S^2–^ oxidation. The gas capture process for the Nesjavellir injection system studied here utilizes liquid ring vacuum pumps located at the condensers to dissolve CO_2_ and H_2_S into geothermal wastewater.^50^ This varies from the CarbFix process of gas capture, which utilizes a pressurized scrubbing tower to dissolve CO_2_ and H_2_S gases.^17,50^ Additional details on the wastewater produced at Nesjavellir, the injection system, and wells NN-3 and NN-4 are included in Supporting Information (SI) Text S1 and Table S1.

### 2.2 Wireline Logging

The geophysical wireline logging methods used to parametrize the reactive transport models and identify H_2_S mineralization are introduced here. These geophysical methods are outlined in red on the study workflow, shown in Figure S1. Logging data (neutron, temperature, resistivity, and IP) collected in the NN-3 and NN-4 injection wells before the start of H_2_S injection (September 21, 2020) define the porosity and permeability parameters used in the geochemical modeling and establish a baseline IP response. Logging measurements were repeated 40 days after the start of injection (March 10, 2021) to evaluate physicochemical changes due to H_2_S injection. Before logging, cold groundwater is injected to decrease the borehole temperature to 60 °C, as required by the logging instruments.

The neutron logging tool emits neutrons from a radioactive americium–beryllium source and uses a neutron receiver to measure the number of neutrons arriving per second.^51^ Values are reported to an American Petroleum Institute industrial standard unit, API. Neutron logs are primarily sensitive to the presence of hydrogen near wells, and reduced neutron responses can indicate high water contents and porosities. However, clays and other hydrous minerals can also lower the neutron response, leading to the misinterpretation of high-porosity zones.^52^

The 16/64 QL40-ELOG/IP probing tool (www.alt.lu/downhole-probes/) measures the electrical resistivity and IP response. It uses the “normal” electrode configuration with a 64-in. electrode spacing, defined as the distance between the current electrode (A) and the potential electrode (N).^53^ The reference potential electrode (M) is located at the surface such that the distance between the potential electrodes (M and N) is considered infinite.

Electrical resistivity (units Ωm) is inversely related to porosity and clay content, with decreasing electrical resistivity values potentially indicating increased porosity and/or clay content. Electrical resistivity is also a function of temperature as defined by Keller and Frischknecht^54^ and Revil et al.^55^ We use this relationship, assuming a temperature coefficient of resistivity of 0.025 °C, to standardize the resistivities to 70 °C. This standardization removes the influence of the geothermal gradient and temperature variations between successive resistivity logs. The influence of temperature on the polarization effect is poorly understood, but it appears to be independent of temperature in samples containing metallic particles over the range of 5–50 °C.^37,55,56^ Therefore, it was not accounted for in the IP data.

The QL40-ELOG/IP instrument also measures time-domain IP. Following the application of an electric field, the instrument records the discharge of polarized materials as a decaying voltage at discrete times.^57,58^ In the presence of metallic particles, the IP response is generally dominated by the volume of polarizable minerals (e.g., pyrite, pyrrhotite, magnetite, and graphite), with larger chargeability responses associated with larger volumes.^29,59,60^ The following IP acquisition parameters were used for each measurement campaign: square-wave current injection (positive-off-negative-off for 2 s each, 8 s total), 450 Hz sampling rate of the voltage during the whole cycle, spatial resolution of 25 cm, and wireline speed of 1.8 m/min.

A common practice is to integrate the decay curve over a definite time interval to obtain the integral chargeability.^29,57,60^ Although the integral chargeability cannot be used for quantitative predictions, it has the advantage of smoothing out the noise and can be comparatively assessed along the borehole. Hereafter, we refer to the integral chargeability as chargeability, *M*, for simplicity.

### 2.3 RTM Chemical Parametrization

The properties of the RTM and the model parametrization are discussed here. These components are outlined in blue in the workflow shown in Figure S1. To assess the processes and physicochemical parameters that control the H_2_S mineralization upon the injection of H_2_S-charged water, 1D advective RTMs were constructed into the adjacent rock along the boreholes using PHREEQC^61^ and the *CarbFix.dat* database.^62,63^ This database is adapted from the *core10.dat* database,^64^ and it includes revised mineral solubilities (including clays and sulfides) and aqueous species stabilities that are specifically tuned to improve the model accuracy of basalt systems at high temperatures (>90 °C).^62,63^

**Figure 1:**
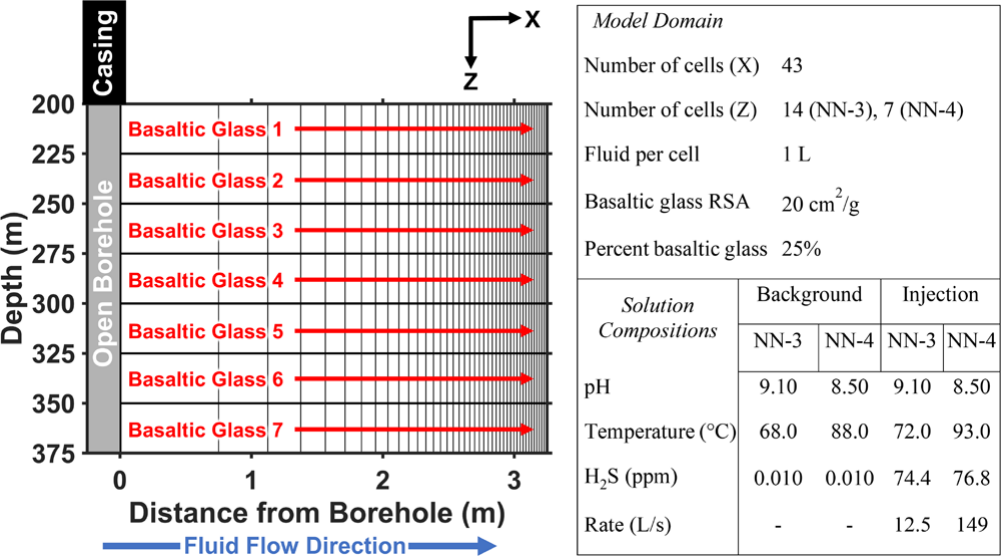
Diagram of the 1D reactive transport model domain (shown for NN-4) and table of the key model parameters. Additional model parameters are listed in Tables S2 and S3. The host rock (basaltic glass) composition is defined for each of the individual flow models, labeled in red. 1D advective flow is along the *x*-direction, and mixing between the individual models is not considered in the modeling. Cell widths are scaled by 1/*r* to model decreases in the radial flow velocity.

In this study, we constructed 1D RTMs that incorporate evolving water chemistry and cumulative basaltic glass dissolution along the lateral flow path, given initial water and rock chemistry representing the H_2_S injection water, basaltic glass kinetic dissolution rates, host rock porosity and permeability, and reaction time step.^65^ The RTM grids, shown in Figure 1, were created by discretizing the NN-3 and NN-4 injection boreholes into layers of 25 m in length. Each layer is treated as an independent 1D RTM. The size and spacing of the grid were based on the availability of chemical data of the host rock and the sensitivity radius of the IP tool (Text S2).^66,67^

The water flow velocities were calculated using Darcy’s law and assuming uniform radial flow. The volumetric flow rate for each layer was determined by scaling the total volumetric injection rate for NN-3 and NN-4 by a mobility factor, which is the permeability of the layer normalized by the total sum of the permeability in the well (Text S3). In PHREEQC, 1D RTMs assume constant velocity along the flow path, so the radial flow velocity was achieved by scaling the cell widths by 1/*r*. These 1D RTMs are beneficial as they can simulate radial flow away from the borehole in a computationally efficient manner, capturing the processes impacting the trends of the bulk geophysical measurements along the borehole.

The basaltic glass host rocks used in the RTMs were represented by the measured bulk composition of drill cuttings collected along the NN-3 and NN-4 boreholes (Table S2). The measured rock compositions were averaged over 25 m-depth intervals (Table S3). The initial background water for each flow model was uniform and had a composition identical with the injection waters measured in NN-3 and NN-4 but with H_2_S depleted (Figure 1 and Table S4). This estimate of the initial water composition represents the geothermal wastewater that has been injected since 2004 and is the same wastewater into which the H_2_S is dissolved into. The H_2_S-charged injection water input into each 1D RTM was assumed to be uniform across each well. The reaction temperature corresponded to an average temperature measured over 1 year for the background water and measured continuously during the 40 days of injection for the H_2_S injection water. Details on sampling and analytical approaches of the bulk rock composition and injection water concentrations are given in SI Text S4.

Similar to previous studies modeling basaltic alteration processes,^1,68−70^ each of the RTMs incorporated kinetically controlled stoichiometric basaltic glass dissolution with the kinetic rate given by Gíslason & Oelkers (2003).^71^ Since the short time frame of H_2_S injection between IP measurements is short (40 days) and basaltic glass dissolution is faster than the dissolution of crystalline minerals in basalt,^72,73^ we assume that basaltic glass is the primary phase affected most by the reaction of the injection fluid and host rock. Basaltic glass is estimated to be 25% of the matrix volume based on measurements from unaltered hyaloclastites in south and southwest Iceland.^74^ The saturation conditions of the dissolving basaltic glass were calculated assuming that the primary phase is a leached layer of amorphous Al-hydroxide and amorphous silica.^75−77^ Its reactive surface area (RSA), a parameter generally unknown in natural settings and uncertain in reactive transport modeling,^78−80^ was assumed to be 20 cm^2^/g of basaltic glass, consistent with previous studies.^25,70^ The RSA varies as a function of the porosity and the amount of basaltic glass dissolved, assuming the dissolution of spherical particles.^81^ The molar volumes of the alteration minerals were taken from Voigt et al. (2018),^62^ and basaltic glass density was assumed to be 2.9 g/cm^3^.^2,76^

Alteration minerals allowed to precipitate at local equilibrium following basaltic glass dissolution were chosen based on alteration mineral assemblages from laboratory studies^1,24,82^ and observations in low-temperature zones within the subsurface of Nesjavellir (Table S5). They include sulfides (pyrite, pyrrhotite), carbonates (calcite, dolomite, magnesite, siderite), zeolites (analcime, thomsonite), clay minerals (celadonite, Ca–K–Na–Al–Mg–Fe–vermiculite, Ca–Na–Mg–saponites), and iron hydroxides (goethite).^46,47,83−86^ Precipitation kinetics for secondary minerals, which are largely unconstrained, are not incorporated into these RTMs. However, laboratory studies showed a strong correlation between the rate of H_2_S mineralization in basaltic systems and the rate of basaltic glass dissolution, suggesting that the basaltic glass dissolution rate is the limiting factor for H_2_S mineralization.^1,3^

To compare the results of the RTMs to the geophysical data, we calculated the volume of the secondary sulfides (pyrite and pyrrhotite) formed per unit volume of formation after the injection of H_2_S-charged water for 40 days. This value is referred to as the sulfide volume fraction (SVF). We then average the change in SVF over the flow paths, weighted by the distance-dependent electric signal contribution.^67^ This provides a single weighted average SVF for each RTM, indicative of the IP response. Hereafter, we refer to this as the IP-equivalent average SVF change. Details on the IP-equivalent average SVF calculations are given in SI Text S2.

### 2.4 RTM Physical Parametrization

**Figure 2:**
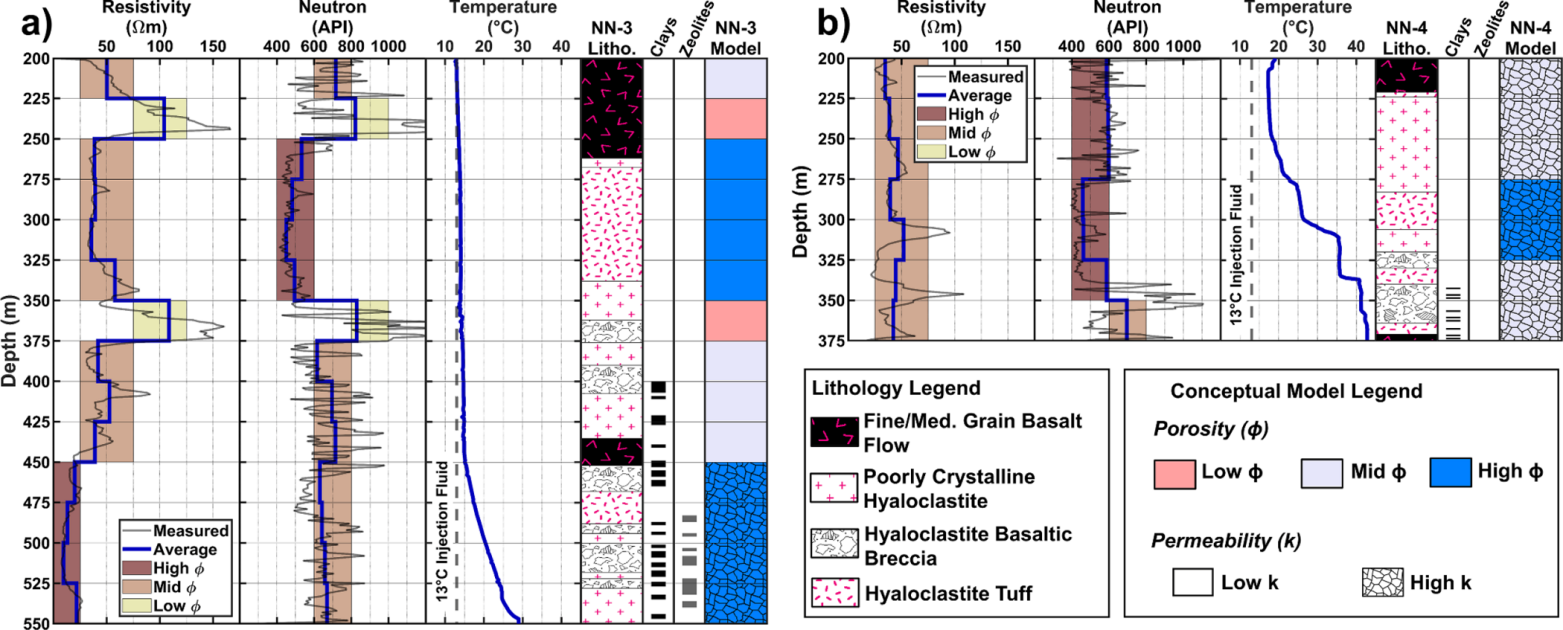
Wireline logging results and drill cutting analysis in NN-3 (a) and NN-4 (b). Wireline logs were collected in September 2020, prior to the start of the H_2_S injection. The average resistivity and neutron response are grouped into categories of 5, 15, and 25% porosity (right columns). The temperature logs used for permeability estimates (1 × 10^–13^ and 1 × 10^–11^ m^2^) were acquired after the cold injection of 13 °C water.^91^

To parametrize the porosity of the RTM, we utilize the electrical resistivity and neutron wireline logs. These wireline logs provide qualitative data on the porosity^52,87^ but have limited ability to define the porosity quantitatively.^87^ Empirically derived relationships for porosity have limited applicability in basaltic aquifers, given their calibration to sedimentary formations and the complex relationships observed between porosity, resistivity, and clay content in basalts.^52,87^ To assign the relative porosity estimates from the resistivity and neutron logs, we group the measured responses into three categories (low–mid–high) based on their average response over 25 m-depth intervals. Resistivity values are grouped as <25, 25–75, and >75 Ωm, and neutron responses are grouped as <600 API, 600–800 API, and >800 API for high, mid, and low porosities, respectively (Figure 2). We assign porosity estimates of 5, 15, and 25% based on literature values reported at Nesjavellir and in the surrounding Hengill area.^88−90^

The permeability values assigned in the RTMs were determined based on temperature logs from 2001, measured 87 and 45 days after the cold injection of 13 °C water into NN-3 and NN-4, respectively.^91^ Temperature changes along the borehole indicate a loss of the cold injection water and inflow of the warmer groundwater, thus highly permeable feed zones. Zones without temperature change indicate low permeability as the injection water does not communicate with the surrounding groundwater.^92^ We focus on broad temperature trends over the 25 m borehole intervals and not localized temperature increases, which may indicate fracture-dominated flow paths that facilitate rapid fluid migration outside the near-borehole model domain. Permeabilities were grouped into high (1 × 10^–11^ m^2^) and low (1 × 10^–13^ m^2^) categories based on values reported for basalts in the Hengill area.^1,70,74,93,94^ These permeability estimates control the injection water supply to each layer in the RTM, with larger permeabilities facilitating more water flow (SI Text S3).

## 3 Results and Discussion

### 3.1 Wireline Results Parametrizing RTMs

Two zones of high porosity are defined in NN-3 from 250 to 350 m depth and 450–550 m depth. In these zones, either the average measured resistivity or neutron response falls into high-porosity grouping. Drill cuttings from these intervals agree with the high-porosity assignment as a vesicular, foam-like porous tuff is observed from 250 to 350 m depth, and secondary alteration minerals are present at the bottom of NN-3 (i.e., clays, zeolites),^49^ indicative of increased water–rock interactions.^2,95^ We assign mid porosity in NN-3 from 200 to 225 and 375 to 450 m depths and low porosity from 225 to 250 and 350 to 375 m depths based on the agreement between the average measured resistivity and neutron responses.

For NN-4, the average measured neutron responses over the 25 m cells are low (<600 API) in the 200–350 m depth range and moderate (∼700 API) in the 350–375 m depth range. Where neutron responses fall near the 600 API threshold value (200–275 and 325–350 m), we assign mid porosities given the moderate resistivities observed throughout the entire well (30–45 Ωm). The neutron response is particularly low from 275 to 325 m depth (∼460 API), so we characterize this interval as high porosity. Additionally, drill cuttings from this interval identify a vesicular, foam-like porous tuff similar to the interval from 250 to 350 m depth in NN-3.^49^

The NN-3 temperature profile is characterized by temperatures close to the injection temperature (<15 °C) down to 450 m. Temperatures start increasing beneath the 450 m depth, indicating increased water flow into the borehole (Figure 2). Therefore, low permeabilities were assigned to the depth range of 200–450 m, and high permeabilities were assigned to 450–550 m. The NN-4 temperature log shows an increase in temperature with depth throughout the entire well. A faster and greater temperature recovery in NN-4 (17–43 °C) compared to NN-3 (13–29 °C) indicates higher water flow into NN-4 compared to NN-3.^91^ Based on this, high permeabilities were assigned in NN-4.

### 3.2 Pyrite Formation Mechanisms upon H_2_S Injection Revealed by RTMs

**Figure 3:**
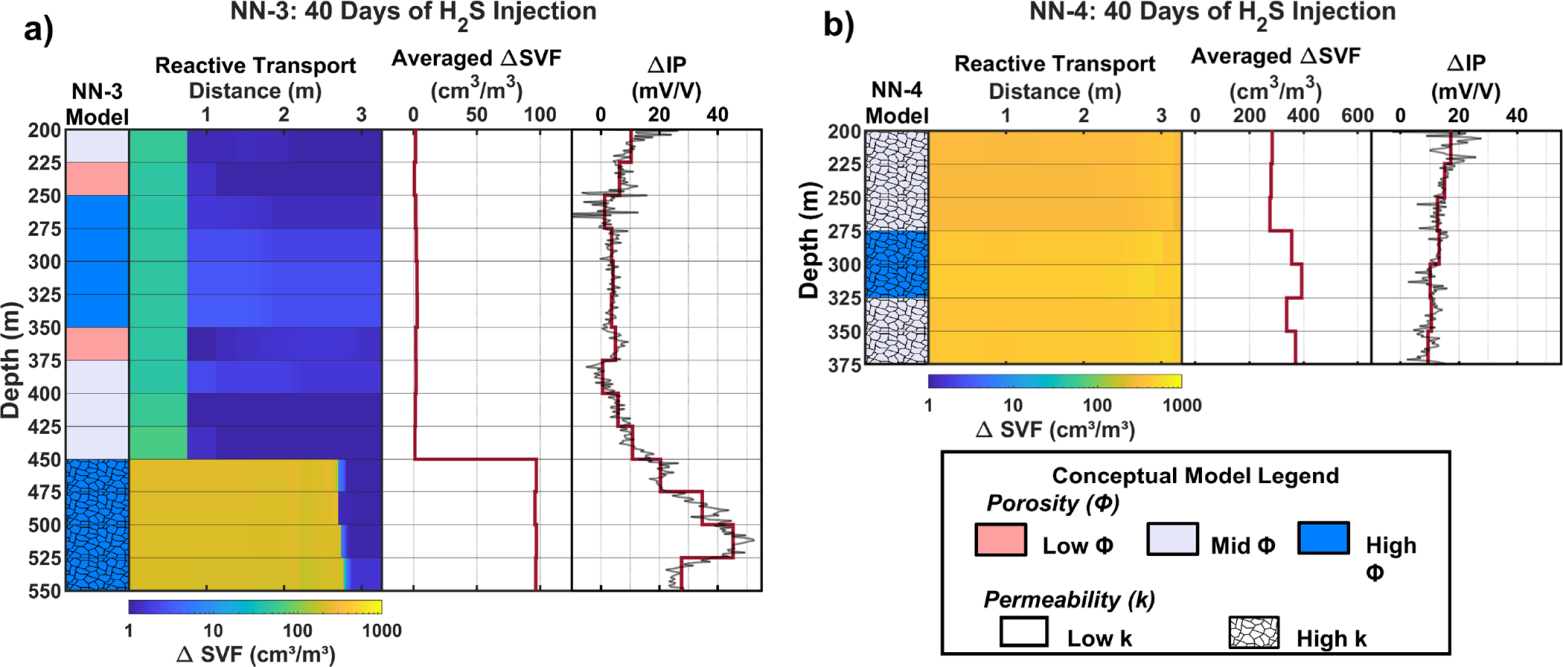
(Left to right) Relative porosity and permeability used in the reactive transport model, together with the change in SVF resulting from the reactive transport modeling along the flow path after 40 days of the H_2_S injection. The change in the IP-equivalent average SVF over the flow path takes account of a weighted function describing the dependence of the electric field magnitude on the distance from the borehole.^67^ The measured change in chargeability (mV/V) after 40 days of H_2_S injection. Results are included for injection wells NN-3 (a) and NN-4 (b).

The results of the RTM show that under the current injection conditions, pyrite, which is supersaturated in the injection water, readily forms in the first cells (Figure 3). Upon basalt dissolution in the H_2_S-charged injection water, the injection-water-sourced S and basalt-sourced Fe allow pyrite formation, following eq 1:
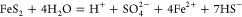
1

The solubility of pyrite is low at the modeled temperature (∼70–90 °C) and pH (8.5–9.1) conditions,^96^ and pyrite formation is favored over other Fe sulfides. Moreover, pyrite formation is found to be controlled by the supply of Fe (from rock leaching) to the system, which agrees with previous laboratory and modeling studies that found Fe availability to be a limiting factor of H_2_S mineralization.^1,2^ Pyrite formation occurs prior to Fe oxides and Fe-bearing smectites, suggesting that pyrite formation is efficient and controlled by the supply of Fe (Figure S2). Additionally, the modeled pyrite formation close to the well at the early stages of the water–rock interaction agrees with field observations of rapid pyrite formation from solids recovered from an airlift pump of an injection well during the Carbfix1 CO_2_–H_2_S injection.^16^

As the reactive water flows through the model, the S supply from the H_2_S-charged water decreases due to pyrite precipitation (Figure S3). Continuous dissolution of basaltic glass and accompanied alteration mineral precipitation ultimately lead to an increase in pH (and decrease in redox potential) over the flow path and favorable conditions for the precipitation of Fe-smectites, goethite, and pyrrhotite. Pyrrhotite, smectite, and goethite formation are most extensive in low-permeability zones, where the water interacts with larger amounts of basaltic glass in each time step (i.e., low water–rock ratios; Figure S2). Zeolite formation is also most extensive at low water–rock ratios.

### 3.3 Coupling RTMs and Wireline Responses to Monitor Subsurface H_2_S Mineralization

Comparing trends in the change in sulfide volume fraction observed in the RTM to the IP results of NN-3 after 40 days of H_2_S injection, we observe a general agreement between the two methods across many intervals of the borehole. The largest change in SVF IP-equivalent average ∼100 cm^3^/m^3^) and the largest increases in the chargeability response (10–50 mV/V) are observed at the bottom of NN-3, where permeability was estimated to be the highest. This suggests that under the current injection conditions of NN-3, sulfide mineralization is controlled largely by permeability, with sulfide formation occurring dominantly in intervals with an increased supply of injection water. Compared to permeability, porosity exhibits less control on sulfide formation in the NN-3 RTM.

In NN-4, the measured chargeability increases over the entire borehole, trending from ∼17 mV/V at 200 m depth to ∼10 mV/V at 375 m depth. The modeled change in SVF in the NN-4 RTM also shows an increase in values over the entire borehole and flow path, supporting the assumption of an evenly distributed water flow (i.e., uniform RTM permeability) throughout the NN-4 intervals. The higher injection rate into NN-4 compared to NN-3 sustains H_2_S mineralization over the entire RTM flow path.

After 40 days of injection, half of the injected H_2_S into NN-4 is mineralized in the near-borehole model. Lower flow rates around NN-3 result in the complete mineralization of the injected H_2_S near the NN-3 borehole over 40 days of injection. This increased localization of H_2_S mineralization around NN-3 relative to NN-4 could help to explain why the largest measured increase in the IP response in NN-3 is 50 mV/V compared with 17 mV/V in NN-4. While these estimates on mineralization efficiency are uncertain due to model simplifications (e.g., no groundwater mixing, single porosity medium), comparing their relative values provides insight into the IP response changes.

The modeled SVF is larger in NN-4 compared to NN-3 due to the larger fluid supply in NN-4. Additionally, higher injection water temperatures in NN-4 compared to NN-3 (93 vs 72 °C) result in faster basalt glass dissolution rates in NN-4, contributing to the higher magnitude changes in SVF near the borehole (Figure S4). The highest SVF change among the first cells is 235 cm^3^/m^3^ in NN-3 (450–475 m), compared to 540 cm^3^/m^3^ in NN-4 (300–325 m).

While agreement between the change in SVF predicted by the RTM and changes in chargeability exists in the intervals detailed above, discrepancies in other intervals illustrate the necessity to further constrain RTM parameters, particularly the porosity and permeability values along the boreholes. A moderate chargeability increase (∼10 mV/V) observed from 200 to 225 m in NN-3 could indicate higher permeability in this zone rather than the low value assigned in the RTM. This is in accordance with ambient temperature logs of NN-3 that identify an inflow of warm water at 200 m, attributed to wastewater disposed of in nearby shallow wells.^91^ In NN-4, the change in IP response slightly decreases with depth while the change in the IP-equivalent average SVF slightly increases with depth. The RTM trend is partially explained by larger porosities in the RTM from 275 to 325 m depth, which increases the reactive surface areas in this interval, leading to increased basaltic glass dissolution and Fe supply. Additionally, variations in the basaltic glass composition contribute more basalt-sourced S and promote faster dissolution rates, resulting in elevated SVF changes near the bottom of NN-4 (Figure S5). However, variations in the basaltic glass compositions have little impact on the H_2_S mineralization efficiency, particularly at early stages of basaltic glass alteration (high water–rock ratios) (Figure S6).

These discrepancies highlight the challenges of using simplified RTMs to quantitatively model system dynamics. First, mixing of water with ambient groundwater is not considered in these RTMs. Mixing could change the water composition and temperature along the borehole, impacting secondary minerals’ kinetic rates and saturation. Next, single porosity advection flow RTMs simplify the hydrologic model and do not capture complex flow structures (e.g., isolated fracture networks and variable tortuosity). However, given the small flow path modeled here and the large fluid flow in proximity to the injection wells, advection-dispersion models are found to produce similar results to the advection-only model (Figure S7). Lastly, reaction rates are challenging to incorporate into RTMs as kinetic precipitation rates are often unconstrained and reactive surface areas are often site-specific values and difficult to determine for field studies.^79,80^ In fact, RTMs utilizing kinetically controlled pyrite precipitation and a range of reactive surface areas from literature^79,97^ recover similar distributions of H_2_S mineralization along the flow path but a 19% difference in the IP-equivalent average SVF (Figure S8). However, we find that the IP-equivalent average SVF change of the equilibrium model used in this study falls between the range of values recovered in the kinetic models, suggesting that the equilibrium-driven precipitation of sulfides is a relevant approximation that adds computational efficiency.

Challenges with linking RTM and IP also arise from complexities in the IP response, such as variable sulfide grain size/shape and clay contributions.^37,57,98^ Influences of metallic mineral grain connectivity and size/shape distributions on chargeability is the subject of ongoing research and the influences are often not considered in the final interpretation.^60,200^ Smectite clay, a common secondary mineral in basaltic rocks at temperatures <100 °C,^84,85,99^ is predicted to precipitate in our model (Figure S2) and can also impact the IP response. Elevated smectite content can decrease the chargeability response as interfoliar current flow increases conduction (reducing resistivity) and decreases the material’s ability to build charge.^37,98^ However, outside of a few isolated zones in NN-3 (225–250 m, 350–375 m, and 400–425 m depths), resistivities remain unchanged since the start of injection, suggesting the limited influence of smectites on the bulk electrical properties upon 40 days of H_2_S injection (Figure S9). Clays can also decrease the neutron response,^52^ and indeed, the average neutron response decreases by 81 API after 40 days of H_2_S injection in NN-3 (Figure S9). However, a similar decrease is not observed in NN-4.

### 3.4 Parameters Influencing Sulfide Formation

#### Physical Parameters and Heterogeneity

**Figure 4:**
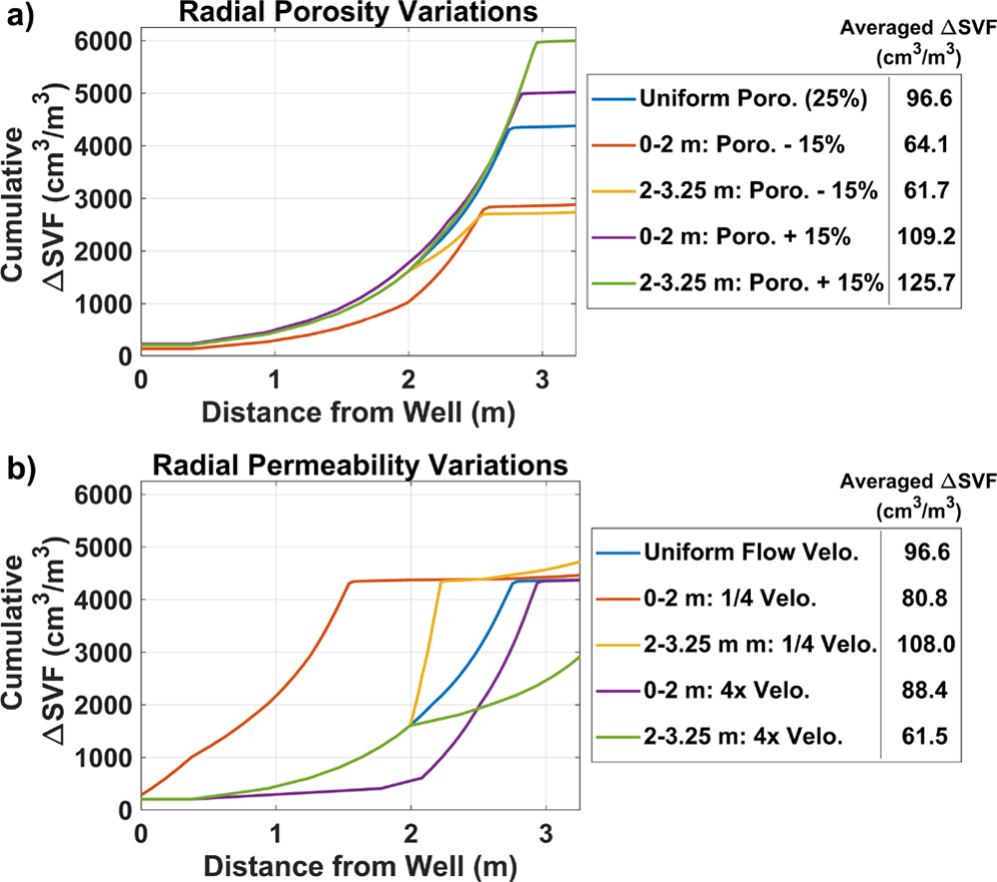
Effects of (a) porosity and (b) permeability heterogeneity on the cumulative change in SVF and the IP-equivalent average SVF change. The porosity and permeability values are adjusted from the 1D reactive transport model of NN-3, 525–550 m depth, which assumed uniform porosity and permeability along the flow path. Changes in the matrix permeability are modeled via the flow velocity.

Additional RTMs were constructed to better understand how radial heterogeneity in porosity and permeability impact the H_2_S mineralization along the flow path and contribute to discrepancies between the RTM results and the measured IP response changes (Figure 4). These models are based on the NN-3 model from 525 to 550 m depths (25% porosity, 1 × 10^–11^ m^2^ permeability), and they utilize different porosity or permeability values assigned from 0 to 2 m and 2 to 3.25 m along the flow path (for example, porosity = 25% from 0 to 2 m and 10% from 2 to 3.25 m).

Porosity heterogeneity was simulated by increasing or decreasing the porosity of the uniform model (25% in NN-3, 525–550 m depth) by 15% while maintaining constant flow velocities. These simulations reveal that the porosity has a minimal influence on the distribution of H_2_S along the flow path (Figure 4). However, larger porosities result in larger cumulative change in SVF, particularly toward the end of the flow path as the water residence times are greater further from the well. The larger SVF changes in the high-porosity system arises from increased RSAs, thereby increasing the supply of basalt-sourced Fe in these high porosity zones. Additionally, larger porosities increase the supply of water-sourced S to the system, thus increasing the total change in SVF over the flow path.

The water flow velocity from the NN-3, 525–550 m depth model (11.7 m/day at the wellbore) is increased and decreased by a factor of 4 to simulate radial heterogeneity in the permeability along the flow path. Due to the short lateral flow path, smaller lateral permeability variations are used here compared to the variations assigned to the field model (Section 2.4) to mimic small variations that occur within the stratigraphic unit. The modeling reveals that permeability heterogeneity influences both the distribution of H_2_S mineralization and the cumulative change in the SVF over the flow path. In the uniform flow velocity model (NN-3, from 525 to 550 m depth), all the water-sourced S is mineralized before 2.75 m along the flow path, indicated by the plateau in the cumulative SVF change at ∼4400 cm^3^/m^3^. Cumulative SVF change greater than 4400 cm^3^/m^3^, observed when flow velocity is low at flow distances of 2 to 3.25 m, is due to H_2_S mineralization from basalt-sourced S resulting from longer water residence times. Decreasing the flow velocity also results in a more rapid H_2_S mineralization along the flow path. Conversely, increasing the water flow velocity at the start of the flow path (0 to 2 m) limits H_2_S mineralization in the first 2 m along the flow path. Large flow velocity from 2 to 3.25 m along the flow path decreases the cumulative change in SVF to below 3000 cm^3^/m^3^ as not all the water-sourced S mineralizes over the 3.25 m flow path. These results suggest that low-permeability zones near the borehole are most at risk of pore clogging due to enhanced secondary mineralization in these zones.

Permeability heterogeneity also impacts the IP-equivalent average of the SVF change and thus likely contributes to discrepancies between the RTM-predicted H_2_S mineralization and the measured IP responses (Figure 3). Since ∼60% of the measured IP signal comes from distances 0.5 to 2 m (Text S2), H_2_S mineralization at this distance range has the most impact on the IP-equivalent average SVF change. For example, most of the H_2_S mineralization occurs at 1 to 2.25 m in the model with reduced flow velocities at 2 to 3.25 m, resulting in the largest IP-equivalent average SVF change of 126 cm^3^/m^3^. Comparatively, the model with reduced flow velocity from 0 to 2 m along the flow path has a lower IP-equivalent average SVF change due to larger amounts of H_2_S mineralization at 0 to 0.5 m, where the IP tool is less sensitive to changes. The models with increased flow rates at 0 to 2 and 2 to 3.25 m also result in lower IP-equivalent average SVF changes as H_2_S mineralization occurs further into the formation.

#### Chemical Parameters

**Figure 5:**
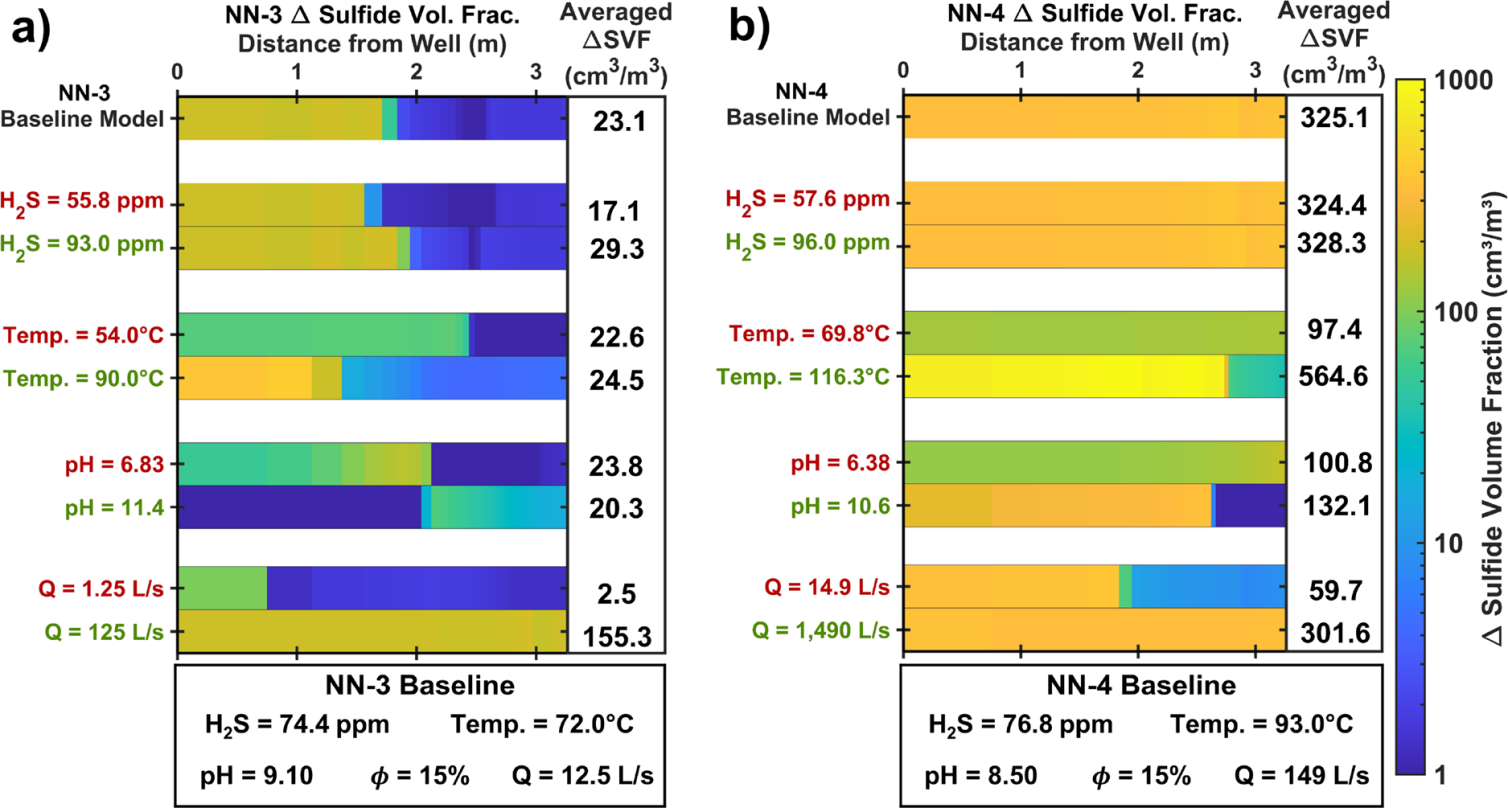
Row 1 of each figure illustrates changes in SVF predicted in the RTM of NN-3 and NN-4 with input data defined in the legend below the plots (measured dissolved H_2_S, pH, and injection water temperature, average basaltic glass composition across the entire borehole, midestimate porosity, and the well injection rates with even allocation of the injection water to each model layer). The additional rows show the change in SVF predicted by RTMs with a single parameter varied, as displayed along the *y*-axis. The chemical parameters are varied by −25% (red) and +25% (green) of the measured values.

To better understand the mechanisms controlling sulfide formation in our RTM, the effects of water chemical parameters (temperature, H_2_S concentration, and pH) on the magnitude and distribution of sulfide formation were investigated (Figure 5). The values are compared to baseline model results, consisting of the measured injection water parameters, 15% porosity (midestimate value), and well injection rates with injection water evenly distributed to each model layer. Overall, the IP-equivalent average change in SVF in NN-3 is less sensitive to parameter changes than that in NN-4. This is because the fluid supply is less in NN-3 compared to that in NN-4, which limits the influence of the fluid chemical parameters on the system.

Variations in the basaltic glass chemical compositions have little influence on the magnitude of the SVF change in NN-3 but influence SVF changes in NN-4 (Figure S5). In NN-4, faster basaltic glass dissolution rates (primarily from higher injection water temperatures) and larger water flow than NN-3 increase the water–rock interactions over the entire flow path. This enhances the influence of basaltic glass composition on the resulting secondary mineralogy.

Changes in the H_2_S concentration of the injection water do not influence sulfide mineralization near the NN-4 borehole (Figure 5). The large flow rates sustain sulfide precipitation over the entire flow path for every modeled H_2_S concentration, suggesting that the basaltic glass dissolution rate (Fe supply) is the limiting factor in the magnitude of H_2_S sequestration in NN-4. In NN-3, increasing the water’s H_2_S concentration by 25% from the baseline value results in a 29% increase in the IP-equivalent average SVF change as sulfides form further into the formation. However, at short flow distances (<1.5 m), the H_2_S concentration does not influence the magnitude of sulfide precipitation. This illustrates that total sulfide mineralization near the NN-3 borehole is controlled by both the water supply and the basaltic glass dissolution rates. The H_2_S itself is found to have minimal influence on the rate of basaltic glass dissolution (<2% rate change) (Figure S4).

The basaltic glass dissolution rates strongly depend on the temperature and pH of the reacting water (Figure S4).^71^ Decreasing the temperature by 25% from the measured values results in slower basaltic glass dissolution and a more limited Fe supply. This decreases the IP-equivalent average sulfide mineralization by 2% in NN-3 and 70% in NN-4. Since the H_2_S supply is larger in NN-4, the limited Fe supply at lower temperatures has a larger relative impact on the IP-equivalent average sulfide mineralization. These model results of decreased H_2_S mineralization at lower temperatures are consistent with lab studies showing more limited H_2_S mineralization at temperatures ∼100 °C compared to higher temperatures (200–250 °C).^1^

The change in the injection water pH from circumneutral to alkaline results in faster basaltic glass dissolution (Figure S4). The faster dissolution reflects the pH dependence of the basaltic glass dissolution rate established in Gislason and Oelkers (2003), with progressively faster rates as pH increases and decreases from pH = 5 at temperatures near 100 °C. However, the faster basaltic glass dissolution from the increased pH does not equate to larger changes in SVF for both boreholes. In NN-3, large pH values of 11.4 (measured pH + 25%) limit sulfide mineralization at the injection inlet, as pyrite is more soluble at this high pH value.^96^ A slightly lower pH of 10.4 in NN-4 enables sulfide mineralization near the injection borehole but at quantities lower than those of the baseline model. This illustrates that the influence of pH conditions on both the dissolution rate and pyrite solubility must be considered to achieve the most efficient mineralization of H_2_S.

#### Well Injection Rate

Changes to the injection water supply greatly control the magnitude of sulfide precipitation along the flow path for both models. For NN-3, an order of magnitude increase in the injection water volumetric rate sustains sulfide precipitation over the entire flow path, thus greatly increasing the IP-equivalent average SVF change (+132 cm^3^/m^3^) from that of the baseline model. This aligns with the findings of the NN-3 RTM (Figure 3), where sulfide mineralization is largely controlled by the water supply (i.e., permeability of each layer and the well injection rate).

Similar to the impact of the H_2_S concentration in NN-4, increasing the injection water supply by an order of magnitude does not increase the IP-equivalent average SVF change in NN-4. The increased water flow results in a slight decrease in the IP-equivalent average SVF change (325 vs 301 cm^3^/m^3^), attributed to slower basaltic glass dissolution from slightly lower pH values maintained over the flow path. The reduced pH results from the fast water flow, which limits the time for water–rock interactions along the flow path. These results further suggest that the Fe supply is the limiting factor in the magnitude of sulfide mineralization when injection rates are large (~hundreds of L/s).

## 4 Implications for IP and RTM Field-Scale Monitoring

Validation of RTM-predicted trends by IP trends observed after 40 days of H_2_S injection suggests that coupling IP wireline logging with RTM can advance the monitoring of H_2_S mineral storage within basaltic reservoirs in Iceland and abroad. The RTMs add spatial and temporal dimensions to the IP survey and provide valuable insight into the parameters influencing the magnitude and distribution of sulfide mineralization.

While RTMs help to understand trends in the IP response, discrepancies between the RTM and geophysical IP responses and the lack of a quantitative link between the SVF and chargeability changes highlight challenges in the current application of this joint monitoring approach. Furthermore, this study does not address the long-term stability of sulfides following H_2_S injection as assessed with subsequent monitoring data presented in Lévy et al. (2024)^100^ To strengthen the joint use of IP and RTM as a monitoring technique and assess the long-term stability of H_2_S mineralization, more complex models are required that incorporate, e.g., measured dissolved oxygen levels to understand how leakage impacts the system, interactions with oxidizing microbes,^101,102^ and retroaction between flow parameters and mineral dissolution/precipitation. Additionally, constraining water flow parameters through direct measurements would reduce uncertainty in the RTM. Lastly, joint geochemical and geophysical monitoring approaches would benefit from a data-based link between site-specific geochemical and geophysical information through direct measurements of the water composition^15,17^ and rock alteration.^103,104^ These measurements reduce critical RTM uncertainties and validate the geophysical responses, which can then be extrapolated over larger areas with expanded RTMs and geophysical surveying. With these additional constraints, joint IP and RTM interpretation represents a scalable, noninvasive, and cost-effective approach for monitoring H_2_S mineralization.

## Data Availability

The data underlying this study are openly available in a Zenodo repository at doi: 10.5281/zenodo.10076311 upon publication.

## Supporting Information Available

(Text S1) Nesjavellir power station wastewater and injection system background; (Text S2) further details on the calculation of the sulfide volume fraction and the weighted averaging function applied to the change in sulfide volume fraction; (Text S3) derivation of the well mobility; (Text S4) complete methodology of the injection water and bulk rock chemical analysis; (Table S1) details on the NN-3 and NN-4 injection wells; (Table S2) basaltic glass whole rock composition; (Table S3) basaltic glass formulas utilized in the RTMs; (Table S4) measured injection water chemistry; (Table S5) chemical formulas of the secondary alteration minerals allowed to precipitate in the RTMs; (Figure S1) the workflow outlining the joint application of geophysical methods and reactive transport modeling to study H_2_S mineralization; (Figure S2) the volume fraction of all secondary minerals from the NN-3 and NN-4 RTMs; (Figure S3) relative source of S upon progressive basaltic glass dissolution; (Figure S4) basaltic glass dissolution rates for the various RTMs; (Figure S5) sulfide formation predicted by RTMs with homogeneous basaltic glass composition; (Figure S6) percentage of total S mineralized upon basaltic glass alteration in reaction path models constructed for all measured basaltic glass compositions in NN-3 and NN-4; (Figure S7) impact of advective transport flow schemes compared to advective-dispersive transport schemes on the change in sulfide volume fraction; (Figure S8) impact of pyrite kinetic precipitation rates on the expected change in sulfide volume fraction compared to RTMs that allow pyrite to mineralize to equilibrium; (Figure S9) and changes in resistivity, neutron, and IP wireline response after 40 days of H_2_S-charged water injection (PDF)

## Abbreviations

Al: aluminum
API: American Petroleum Institute industrial standard unit
Ca: calcium
CO_2_: carbon dioxide
Fe: iron
H_2_S: hydrogen sulfide
IP: induced polarization
K: potassium
M: chargeability
Mg: magnesium
Na: sodium
RSA: reactive surface area
RTM: reactive transport model
S: sulfur
SVF: sulfide volume fraction
1D: one-dimensional
