# Supplementary material for: Advanced Monitoring of H2S Injection through the Coupling of Reactive Transport Models and Geophysical Responses
Source: Environ Sci Technol. 2024 Jun 10;58(25):11128–39. doi: 10.1021/acs.est.3c10139 (PMC11210474; doi:10.1021/acs.est.3c10139)
Supplement: Supplementary file 1 — es3c10139_si_001.pdf [file es3c10139_si_001.pdf]

Supporting Information for:

Advanced monitoring of H_2S injection through the coupling of reactive transport models and geophysical responses

Daniel A. Ciraula[1,*], Barbara I. Kleine-Marshall[2], Iwona M. Galeczka[3,4], Léa Lévy[3,5]

[1]Nordic Volcanological Center, Institute of Earth Sciences, University of Iceland, 101 Reykjavík, Iceland
[2]GeoZentrum Nordbayern, Friedrich-Alexander-Universität, Schlossgarten 5, 91054 Erlangen-Nuremberg, Germany
[3]ÍSOR-Iceland Geosurvey, Urðarhvarf 8, 203 Kópavogur
[4]Carbfix, Höfðabakki 9D, 110 Reykjavík, Iceland
[5]Lund University, Engineering Geology, Lund Box 117, SE-221 00, Sweden
*Correspondence author at: Nordic Volcanological Center, Institute of Earth Sciences, University of Iceland, 101 Reykjavík, Iceland. E-mail address: danielac@hi.is (D.A. Ciraula).

Summary: 24 pages, 4 text descriptions, 5 tables, 9 figures

Supporting text (pages S2-S8) providing (Text S1) background on the wastewater produced and the injection system at Nesjavellir power station; (Text S2) details on the application of the weighed averaging function derived from the induced polarization logging tool sensitivity applied to the sulfide volume fraction over the 1-dimensional flow path; (Text S3) the derivation of the well mobility factor used to determine the volume rate of injection water entering each model layer; (Text S4) a complete description of the injection fluid and bulk rock chemical analysis methods.

Tables (pages S9-S13) include data on (Table S1) the NN-3 and NN-4 injection wells; (Table S2) measured basalt whole rock composition; (Table S3) basaltic glass formulas for the reactive transport models (RTMs); (Table S4) measured injection water chemistry; (Table S5) and the chemical compositions of the secondary alteration minerals allowed to precipitate in the RTMs.

Figures (pages S14-S22) displaying (Figure S1) The workflow outlining the joint application of geophysical methods and reactive transport modeling to study H_2S mineralization; (Figure S2) The volume fraction of all secondary minerals from the NN-3 and NN-4 RTMs; (Figure S3) relative source of S upon progressive basaltic glass dissolution; (Figure S4) basaltic glass dissolution rates for the various RTMs; (Figure S5) sulfide formation predicted by RTMs with homogeneous basalt composition; (Figure S6) percentage of total S mineralized upon basaltic glass alteration in reaction path models constructed for all measured basalt compositions in NN-3 and NN-4; (Figure S7) impact of advective transport flow schemes compared to advective-dispersive transport schemes on the change in sulfide volume fraction; (Figure S8) impact of pyrite kinetic precipitation rates on the expected change in sulfide volume fraction compared to RTMs that allow pyrite to mineralize to equilibrium; (Figure S9) changes in resistivity, neutron, and IP wireline response after 40 days of H_2S-charged water injection.

References for supporting information (pages S23-S24) (PDF)

Text S1: Nesjavellir Wastewater and Injection System

The Nesjavellir power plant produces three types of wastewater. First is separated geothermal water (SGW), which is the aqueous phase of the high enthalpy production fluid. Second is condensate water, which is the steam from the high enthalpy production fluid used to turn the turbines. Third is cooling water, which is cold groundwater used to condense the steam.

A controlled pressure decrease of the utilized geothermal fluid produces steam and separated geothermal water (SGW) at 195°C. The operation of all four turbines for electricity generation at Nesjavellir requires 240 kg/s of steam. To cool the steam, condensers require ~2,000 l/s of cold groundwater (5-7°C). The condensed steam forms the condensate wastewater, composed of dilute water with some dissolved gas (primarily CO_2 and H_2S). The cold groundwater leaves the condensers at around 55°C and is either sent to cooling towers in preparation for disposal or sent to heat exchanges for further usage. The heat exchangers use the 195°C SGW to heat the groundwater to 87°C, where it is then pumped 27 km to Reykjavík.

After steam production from the geothermal production fluid, the remaining SGW contains high concentrations of dissolved solids, posing a considerable risk of both thermal and chemical pollution of surface waters. For this reason, SGW is diluted with the condensate wastewater and injected into the subsurface, including the NN-3 and NN-4 injection wells investigated in this study. Excess SGW and condensate water is discharged at the surface. Much of the SGW is pumped through a delay tank before injection where the dissolved silica polymerizes limiting amorphous silica scaling formation within the injection well and the injection reservoir. However, some amount of untreated SGW is sent to the injection wells, except for NN-3 which only receives SGW from the delay tank.

H_2S and CO_2 gases are captured from the condensers at the power plant using liquid ring vacuum pumps. This differs from the CarbFix process, which uses scrubbing towers to dissolve CO_2 and H_2S. This results in larger concentrations of CO_2 and H_2S compared to the injection waters of CO_2 (1,220 ppm) and H_2S (481 ppm) compared to this study (CO_2 ~10 ppm, H_2S ~75 ppm) (Galeczka et al., 2022). Gas-charged water from the CarbFix process is sent to a deeper injection well at Nesjavellir (NJ-18) (Galeczka et al., 2022). In the shallow reinjection system studied here, the captured gases are dissolved into the vacuum pump seal water (lásvatn), composed of heated groundwater or condensate wastewater. Starting in January of 2021, the seal

water has been mixed with the condensate and SGW, constituting the final, H_2S-charged injection fluid. Prior to January 2021, the injection fluid for NN-3 and NN-4 was composed of condensate and SGW only, and thus had lower concentrations of dissolved H_2S and CO_2.

Text S2: Calculations of Sulfide Volume Fraction and the Weighted Average

In the PHREEQC advection model, the user specifies the cell residence time rather than cell geometry and flow parameters (i.e., permeability). For each simulation, the chemical reactions are calculated relative to a representative volume (RV) that contains 1 L of water when fully saturated. PHREEQC returns the moles of secondary minerals mineralized per L of water. Therefore, since the volume of water in the system is fixed at 1L, changes in the porosity ($\emptyset$) change the representative volume. Additionally, the initial moles of solid reactants are scaled by the factor of (1-porosity)/porosity to account for porosity variability in the starting model.

$$RV = {1{,}000\ cm^3}/{\emptyset}$$

To recover the volumetric proportion of sulfide minerals in each cell (i.e., sulfide volume fraction SVF), we divide the volume of sulfides precipitated (pyrite and pyrrhotite) by the cell's representative porous-medium volume, which factors in porosity variability. This SVF term is equal to the change in SVF as we assume there are no sulfides in the host rock.

$$SVF = \frac{V_{sulfides}}{RV} = \frac{moles\ per\ L\ water_{sulfides} \times molar\ volume_{sulfides}}{RV}$$

To best compare the results of the reactive transport model to the IP wireline results, we average the change in sulfide volume fraction over the entire flow path after 40 days to obtain a single representative value for the given depth interval. We apply a weighted average to account for varying contributions to the measured IP signal based on the distance of H_2S mineralization away from the borehole. The contribution function is formulated in Roy & Dhar (1971), and considers electrostatic potentials of individual cylindrical shells of varying radii integrated to obtain the total contribution to the measured signal. For a normal sonde, as used in this study, the radial investigation characteristic (*RIC*) is defined as follows:

$$RIC = \frac{\rho I}{8\pi} dr \cdot \int_{-\infty}^{+\infty} \frac{r^3 + rz(z-L)}{(r^2+z^2)^{3/2}[r^2+(z-L)^2]^{3/2}} dz\ ,$$

where ρ is the resistivity, I is the current, dr is the thickness of the circular ground element, r is the average radius of the circular ground element, z is the cell height variable, and L is the electrode spacing (64"). Using the wireline resistivity and current measurements, we solve for the RIC relative contribution over 0.5 m bins from 0 to 3.5 m. The RIC values are normalized to 90% of the total signal, given that 90% of the signal is expected over 3.25 m (2 times the electrode spacing) (Roy & Dhar, 1971). These contributions are shown in the following figure. For every bin, *i*,

ranging from x = a to b away from the borehole center, the SVF for all cells located within that bin (taking into consideration the radius of the borehole) are totaled and scaled by the relative contribution of the bin:

$$\Delta SVF_i = RIC_i \times \sum_{x=a}^{x=b} \Delta SVF.$$

Finally, the average change in sulfide volume fraction ($\bar{x}_{\Delta SVF}$) is calculated by multiplying each bin's SVF change by the volume of each 0.5 m bin, given the bin's width ($r_{i,2} - r_{i,1}$), relative to the volume of the total flow column away from the borehole (3.25 m + 0.2 m borehole radius). These resulting SVF contributions are summed over the flow path.

$$\bar{x}_{\Delta SVF} = \sum_{i=1}^{7} \Delta SVF_i \times \frac{{r_{i,2}}^2 - {r_{i,1}}^2}{3.45^2}.$$

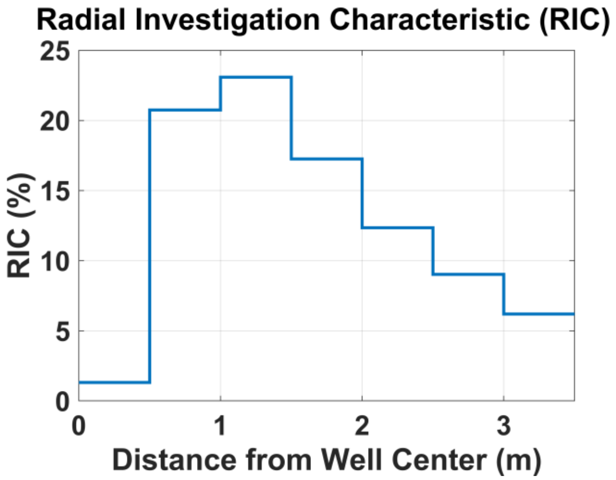

The radial investigation characteristic showing the relative contribution to the IP signal as a function of distance from the center of the borehole (Roy & Dhar, 1971). The signal is grouped into 0.5 m bins, and the total volume of sulfides that mineralize in the bin is scaled by the RIC of that bin.

Text S3: Injection Mobility Factor

The injection mobility factor (μ) is the relative proportion of the total injection fluid (Q_T) that enters each RTM layer (i) along the injection wells (Kipp, 1987). It is derived from Darcy's Law relating the volumetric fluid flux (Q) to the hydraulic conductivity (K), the cross-sectional flow area (A), and the hydraulic gradient ($\partial h/\partial x$). We solve for the relative volumetric fluid flux for n layers as

$$\mu_i = \frac{Q_i}{Q_T} = \frac{-K_i * A_i * \frac{\partial h_i}{\partial x}}{\sum_{i=1}^{n} -K_i * A_i * \frac{\partial h_i}{\partial x}} .$$

Given that all layers have equal cell height (h) and assuming the hydraulic gradient is uniform across all layers, the pressure differences are independent of depth, and the borehole has the same radius (r) across the entire domain, the relationship above simplifies as follows:

$$\mu_i = \frac{-K_i * 2\pi rh * \frac{\partial h_i}{\partial x}}{\sum_{i=1}^{n} -K_i * 2\pi rh * \frac{\partial h_i}{\partial x}} = \frac{K_i}{\sum_{i=1}^{n} K_i}$$

Further assuming that injection fluid density and dynamic viscosity are uniform across the entire well, the hydraulic conductivity ratio becomes equivalent to the ratio of permeability (k),

$$\mu_i = \frac{K_i}{\sum_{i=1}^{n} K_i} = \frac{k_i}{\sum_{i=1}^{n} k} .$$

Text S4: Injection fluid and bulk rock chemical analyses

The chemical composition of the injection fluid and bulk rock from drill cuttings was analyzed to investigate the chemical heterogeneity of the host rock and injection fluid as well as other physiochemical parameters (e.g., pH, injection rate, temperature) on the extent of H_2S mineralization upon injection.

Injection fluid samples (n = 2) were sampled at the wellhead of NN-3 and NN-4 injection wells in November 2022. The temperature and electrical conductivity were measured on-site. The pH of the fluid samples was analyzed at 21°C using a pH meter and electrode calibrated with commercial buffer solution (analytical precision ±0.1). Fluid samples for major anion and cation analyses were prepared as described in Arnórsson et al. (2006). Analyses were carried out at the Institute of Earth Sciences, University of Iceland, using an Inductively Coupled Plasma Optical Emission Spectroscopy (ICP-OES) instrument (ThermoFisher iCAP 7400 Duo) for cations and an ion chromatography (IC) instrument (Dionex ICS-2000) for anions. Dissolved sulfide (H_2S) concentrations were measured via mercury acetate titration with dithizone as the indicator (Arnórsson et al., 2006). Carbon dioxide (CO_2) concentrations were measured through modified alkalinity titration (Arnórsson et al., 2006; Jeffery et al., 1989; Stefánsson et al., 2007) as follows,

$$CO_2(ppm) = \frac{(mL\ HCl \times Molarity\ HCl - mL\ NaOH \times Molarity\ NaOH)}{mL\ sample} \times 44{,}000 - 1.182 \times H_2S(ppm).$$

The analytical precision of major elements was based on duplicate analysis of the samples and was found to be <5% at the 95% confidence level in all cases.

The injection fluids are found to be alkaline (pH > 7) for both NN-3 and NN-4, with the NN-4 fluid having a slightly higher pH than the NN-3 fluid (9.1 vs. 8.5). The NN-3 and NN-4 injection fluids have similar chemical compositions, particularly in measured H_2S concentration (NN-3 = 74.4 ppm and NN-4 = 76.8 ppm). Compared to meteoric water reported at the nearby Vellankatla natural spring (Gysi & Stefánsson, 2012), the injection fluids are generally dilute but with elevated H_2S (NN-3 = 74.4 ppm and NN-4 = 76.8 ppm), SiO_2 (NN-3 = 435 ppm and NN-4 = 428 ppm) and K (NN-3 = 20.3 ppm and NN-4 = 20.3 ppm) concentrations.

Bulk rock compositions from drill cuttings (n = 28) were collected from beneath the casing at the NN-3 and NN-4 injection wells. All samples were milled to a grain size of <150 μm. Total carbon (C) and sulfur (S) analyses of the drill cuttings were conducted using an ELTRA combustion infrared carbon and sulfur analyzer (www.actlabs.com/geochemistry). The

data error range was taken from the detection limit of the equipment for all species analyzed (0.01 wt.%). For major component analyses (SiO_2, Al_2O_3, FeO, MnO, MgO, CaO, Na_2O, K_2O, TiO_2) via ICP-OES, a subset of the milled samples (n = 10) was fluxed with LiB_2 at 1,000°C for 30 min, and subsequently digested in a solution of 5.0 vol% nitric acid (16 molar), 1.3 vol% hydrochloric acid (12 molar), 1.3 vol% oxalic acid (0.8 molar). Accuracy and precision of the analyses were ensured by duplicate sample analyses and repeated analyses of the United States Geological Survey (USGS) standard reference material (BHVO-1 and W-2; Jochum et al., 2016). We interpolated the major element compositions at every total C and S measurement and renormalized the wt.% to account for the C and S compositions not measured via ICP-OES.

Table S1: NN injection well information

Table S1: Details on the Nesjavellir NN injection wells in this study.

Well ID	NN-3	NN-4
Coordinates[a]	64°06’38.23” N, 21°15’20.27” W	64°06’50.03” N, 21°15’0.06” W
Measured Depth (m)[b,c]	563	422
Deviation[b,c]	0°	0°
Casing Depth (m)[b,c]	205	201
Feed Zone Depths (m)[a]	390, 445, 535	280, 300, 330, 388
Flow Rate (kg/s)	12.5	149

[a] Gómez-Díaz et al. (2022), [b] Hafstað et al. (2015), [c] Hafstað (2003)

Table S2: Rock Chemistry

Table S2: Rock compositions measured via ICP-OES and IR-CSA for the NN-3 and NN-4 H_2S injection wells.

Depth (m)	SiO_2	Al_2O_3	FeO	MnO	MgO	CaO	Na_2O	K_2O	TiO_2	C[a]	S[a]
Well NN-3 (wt.%)											
206[b]	48.52	14.83	11.95	0.20	7.46	12.20	2.15	0.19	1.94	0.54	0.03
220	49.38	14.81	12.12	0.20	7.01	11.68	2.18	0.28	2.09	0.27	< 0.01
236	50.29	14.75	12.29	0.20	6.48	11.06	2.21	0.37	2.25	0.10	< 0.01
254	51.04	14.61	12.43	0.21	5.85	10.31	2.24	0.47	2.42	0.41	0.01
272[b]	52.04	14.54	12.62	0.21	5.25	9.60	2.28	0.58	2.60	0.24	0.03
290	52.36	14.44	12.61	0.22	4.95	9.19	2.53	0.69	2.61	0.37	0.04
310	52.84	14.36	12.62	0.23	4.64	8.75	2.81	0.82	2.63	0.26	0.05
332	53.26	14.25	12.60	0.25	4.28	8.25	3.12	0.95	2.65	0.35	0.05
348[b]	53.67	14.19	12.62	0.26	4.03	7.90	3.34	1.05	2.67	0.22	0.06
376	52.08	14.20	12.90	0.25	4.88	9.15	3.05	0.76	2.55	0.15	0.03
400	50.67	14.20	13.14	0.24	5.61	10.21	2.79	0.50	2.45	0.18	0.01
426[b]	49.17	14.20	13.40	0.23	6.40	11.36	2.51	0.23	2.34	0.15	< 0.01
452	48.83	14.37	13.05	0.23	6.50	11.64	2.52	0.28	2.30	0.27	< 0.01
480	48.47	14.56	12.68	0.23	6.61	11.94	2.52	0.33	2.26	0.41	< 0.01
510[b]	48.23	14.80	12.32	0.24	6.74	12.30	2.53	0.39	2.22	0.24	< 0.01
540	48.86	14.99	11.89	0.21	7.22	12.18	2.26	0.25	1.87	0.24	0.03
562[b]	49.34	15.14	11.58	0.20	7.57	12.11	2.07	0.14	1.61	0.22	0.03
Well NN-4 (wt.%)											
206[b]	50.06	16.41	10.13	0.16	5.96	12.54	2.34	0.20	2.02	0.17	< 0.01
220	49.90	16.32	10.02	0.16	6.33	12.75	2.26	0.18	1.83	0.25	< 0.01
236	49.86	16.26	9.92	0.16	6.78	13.02	2.17	0.15	1.62	0.05	< 0.01
254	49.64	16.14	9.77	0.16	7.26	13.28	2.06	0.12	1.37	0.17	0.01
272[b]	49.44	16.03	9.63	0.17	7.73	13.55	1.96	0.09	1.12	0.27	< 0.01
290	49.18	15.66	10.26	0.18	7.39	13.13	2.09	0.17	1.42	0.47	0.04
310	49.21	15.35	11.02	0.20	7.05	12.75	2.26	0.25	1.76	0.08	0.06
332	48.92	14.92	11.79	0.22	6.63	12.24	2.42	0.34	2.13	0.34	0.05
354[b]	48.81	14.54	12.60	0.24	6.24	11.79	2.60	0.43	2.50	0.23	0.02
376	49.11	14.51	12.22	0.23	6.54	12.19	2.45	0.36	2.27	0.12	0.01
388[b]	49.17	14.46	11.99	0.22	6.69	12.39	2.36	0.32	2.13	0.25	0.02

[a] Measured via IR-CSA.

[b] Samples measured with ICP-OES. Otherwise, interpolated between measurements and renormalized given IR-CSA measured C and S wt.%.

Table S3: Basaltic Glass Phases for RTM

Table S3: Basaltic glass compositions defining the primary phase of the RTMs for wells NN-3 and NN-4.

Depth (m)	Basaltic Glass Formula
Well NN-3	
200-225	$Si_{1.00}Al_{0.357}Fe_{0.2055}Mn_{0.00343}Mg_{0.221}Ca_{0.261}Na_{0.0857}K_{0.00612}Ti_{0.031}C_{0.0416}S_{0.00058}O_{3.45}$
225-250	$Si_{1.00}Al_{0.346}Fe_{0.2042}Mn_{0.00342}Mg_{0.192}Ca_{0.236}Na_{0.0854}K_{0.0094}Ti_{0.0336}C_{0.00995}S_{0.000}O_{3.32}$
250-275	$Si_{1.00}Al_{0.333}Fe_{0.2038}Mn_{0.00342}Mg_{0.161}Ca_{0.207}Na_{0.085}K_{0.0131}Ti_{0.0366}C_{0.0317}S_{0.000724}O_{3.30}$
275-300	$Si_{1.00}Al_{0.325}Fe_{0.2011}Mn_{0.00358}Mg_{0.141}Ca_{0.188}Na_{0.0936}K_{0.0168}Ti_{0.0375}C_{0.0354}S_{0.00143}O_{3.26}$
300-325	$Si_{1.00}Al_{0.32}Fe_{0.1996}Mn_{0.00376}Mg_{0.131}Ca_{0.177}Na_{0.103}K_{0.0197}Ti_{0.0375}C_{0.0246}S_{0.00177}O_{3.22}$
325-350	$Si_{1.00}Al_{0.314}Fe_{0.1978}Mn_{0.00403}Mg_{0.116}Ca_{0.162}Na_{0.117}K_{0.0239}Ti_{0.0374}C_{0.0267}S_{0.00193}O_{3.19}$
350-375	$Si_{1.00}Al_{0.317}Fe_{0.2023}Mn_{0.00409}Mg_{0.126}Ca_{0.173}Na_{0.117}K_{0.0218}Ti_{0.0371}C_{0.0175}S_{0.00159}O_{3.20}$
375-400	$Si_{1.00}Al_{0.321}Fe_{0.2071}Mn_{0.00407}Mg_{0.14}Ca_{0.188}Na_{0.113}K_{0.0185}Ti_{0.0368}C_{0.0144}S_{0.00108}O_{3.23}$
400-425	$Si_{1.00}Al_{0.33}Fe_{0.2173}Mn_{0.00404}Mg_{0.165}Ca_{0.216}Na_{0.107}K_{0.0127}Ti_{0.0363}C_{0.0178}S_{0.00037}O_{3.30}$
425-450	$Si_{1.00}Al_{0.34}Fe_{0.2279}Mn_{0.004}Mg_{0.194}Ca_{0.248}Na_{0.0991}K_{0.00595}Ti_{0.0358}C_{0.0153}S_{0.000}O_{3.38}$
450-475	$Si_{1.00}Al_{0.347}Fe_{0.2235}Mn_{0.00404}Mg_{0.198}Ca_{0.255}Na_{0.0999}K_{0.00729}Ti_{0.0355}C_{0.0277}S_{0.000}O_{3.42}$
475-500	$Si_{1.00}Al_{0.354}Fe_{0.2189}Mn_{0.00408}Mg_{0.203}Ca_{0.264}Na_{0.101}K_{0.00876}Ti_{0.0351}C_{0.0423}S_{0.000}O_{3.47}$
500-525	$Si_{1.00}Al_{0.362}Fe_{0.2132}Mn_{0.00413}Mg_{0.208}Ca_{0.273}Na_{0.102}K_{0.0104}Ti_{0.0347}C_{0.0249}S_{0.000}O_{3.45}$
525-550	$Si_{1.00}Al_{0.362}Fe_{0.2038}Mn_{0.0037}Mg_{0.22}Ca_{0.267}Na_{0.0898}K_{0.00648}Ti_{0.0288}C_{0.0246}S_{0.00115}O_{3.43}$
Well NN-4	
200-225	$Si_{1.00}Al_{0.386}Fe_{0.1692}Mn_{0.0027}Mg_{0.184}Ca_{0.271}Na_{0.0892}K_{0.00492}Ti_{0.029}C_{0.0211}S_{0.000}O_{3.38}$
225-250	$Si_{1.00}Al_{0.384}Fe_{0.1665}Mn_{0.00275}Mg_{0.203}Ca_{0.28}Na_{0.0844}K_{0.00396}Ti_{0.0244}C_{0.00502}S_{0.000}O_{3.36}$
250-275	$Si_{1.00}Al_{0.383}Fe_{0.1646}Mn_{0.00283}Mg_{0.226}Ca_{0.291}Na_{0.0787}K_{0.00283}Ti_{0.0189}C_{0.0222}S_{0.000189}O_{3.41}$
275-300	$Si_{1.00}Al_{0.375}Fe_{0.1742}Mn_{0.00313}Mg_{0.224}Ca_{0.286}Na_{0.0825}K_{0.00434}Ti_{0.0217}C_{0.0478}S_{0.00152}O_{3.47}$
300-325	$Si_{1.00}Al_{0.368}Fe_{0.1874}Mn_{0.00344}Mg_{0.214}Ca_{0.278}Na_{0.089}K_{0.00647}Ti_{0.027}C_{0.00813}S_{0.00229}O_{3.39}$
325-350	$Si_{1.00}Al_{0.359}Fe_{0.2012}Mn_{0.00378}Mg_{0.202}Ca_{0.268}Na_{0.0961}K_{0.00881}Ti_{0.0327}C_{0.0348}S_{0.00192}O_{3.44}$
350-375	$Si_{1.00}Al_{0.351}Fe_{0.216}Mn_{0.00413}Mg_{0.191}Ca_{0.259}Na_{0.103}K_{0.0112}Ti_{0.0386}C_{0.0236}S_{0.000768}O_{3.42}$

Table S4: Fluid Chemistry

Table S4: Injection fluid composition measured via ICP-OES and IC analysis. Fluids were sampled at the injection well heads.

Well no.	NN-3	NN-4
Fluid (ppm)		
pH	9.10	8.50
Cond. (µS/cm)[a]	637	678
Temperature (°C)[b]	64.0	85.0
H_2S	74.4	76.8
SO_4	8.56	10.4
CO_2	13.5	8.36
SiO_2	435	428
Na	97.3	97.4
K	20.3	20.3
Ca	0.420	0.420
Mg	0.100	0.0960
Fe	0.0150	0.0100
Al	1.13	1.13
B	1.11	1.12
Cl	88.4	88.1
F	0.760	0.760

[a] Measured in the field at the wellhead. Otherwise, measured in the lab at 21°C.
[b] Averaged from continuous measurements since start of H_2S injection

Table S5: Secondary RTM Phases

Table S5: Secondary minerals included in the geochemical RTMs for wells NN-3 and NN-4.

Minerals	Chemical Composition
Secondary Phases	
Analcime	$Na_{0.96}Al_{0.96}Si_{2.04}O_6{:}1H_2O$
Calcite	$CaCO_3$
Celadonite	$KMgAlSi_4O_{10}(OH)_2$
Dolomite	$CaMg(CO_3)_2$
Goethite	$FeOOH$
Magnesite	$MgCO_3$
Pyrite	FeS_2
Pyrrhotite	FeS
Vermiculite-Ca-Mg-Al	$Ca_{0.4}Mg_{2.5}Al_{1.8}Si_{2.7}O_{10}(OH)_2$
Vermiculite-Ca-Mg-Fe	$Ca_{0.4}Mg_{2.5}Fe_{0.5}Al_{1.3}Si_{2.7}O_{10}(OH)_2$
Vermiculite-K-Mg-Al	$K_{0.8}Mg_{2.5}Al_{1.8}Si_{2.7}O_{10}(OH)_2$
Vermiculite-K-Mg-Fe	$K_{0.8}Mg_{2.5}Fe_{0.5}Al_{1.3}Si_{2.7}O_{10}(OH)_2$
Vermiculite-Mg-Mg-Al	$Mg_{2.9}Al_{1.8}Si_{2.7}O_{10}(OH)_2$
Vermiculite-Mg-Mg-Fe	$Mg_{2.9}Fe_{0.5}Al_{1.3}Si_{2.7}O_{10}(OH)_2$
Vermiculite-Na-Mg-Al	$Na_{0.8}Mg_{2.5}Al_{1.8}Si_{2.7}O_{10}(OH)_2$
Vermiculite-Na-Mg-Fe	$Na_{0.8}Mg_{2.5}Fe_{0.5}Al_{1.3}Si_{2.7}O_{10}(OH)_2$
Saponite-Mg-Ca	$Ca_{0.175}Mg_3Al_{0.35}Si_{3.65}O_{10}(OH)_2$
Saponite-Mg-Na	$Na_{0.35}Mg_3Al_{0.35}Si_{3.65}O_{10}(OH)_2$
Siderite	$FeCO_3$
Thomsonite	$Ca_2NaAl_5Si_5O_{20}{:}6H_2O$

*Average composition of all measured samples

Figure S1: Study Workflow

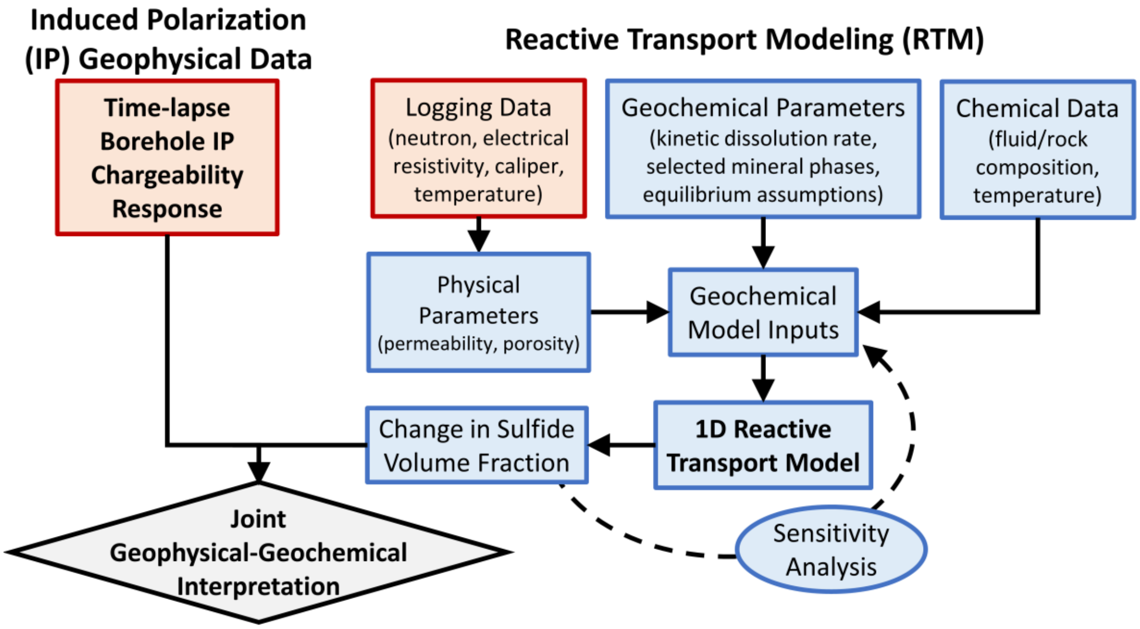

Figure S1. The workflow deployed in this study where the IP geophysical responses and reactive transport models were used to track the magnitude and distribution of H_2S mineralization upon injection. Geophysical and geochemical modeling methods are shown in red and blue boxes, respectively.

Figure S2: RTM Secondary Mineral Formation

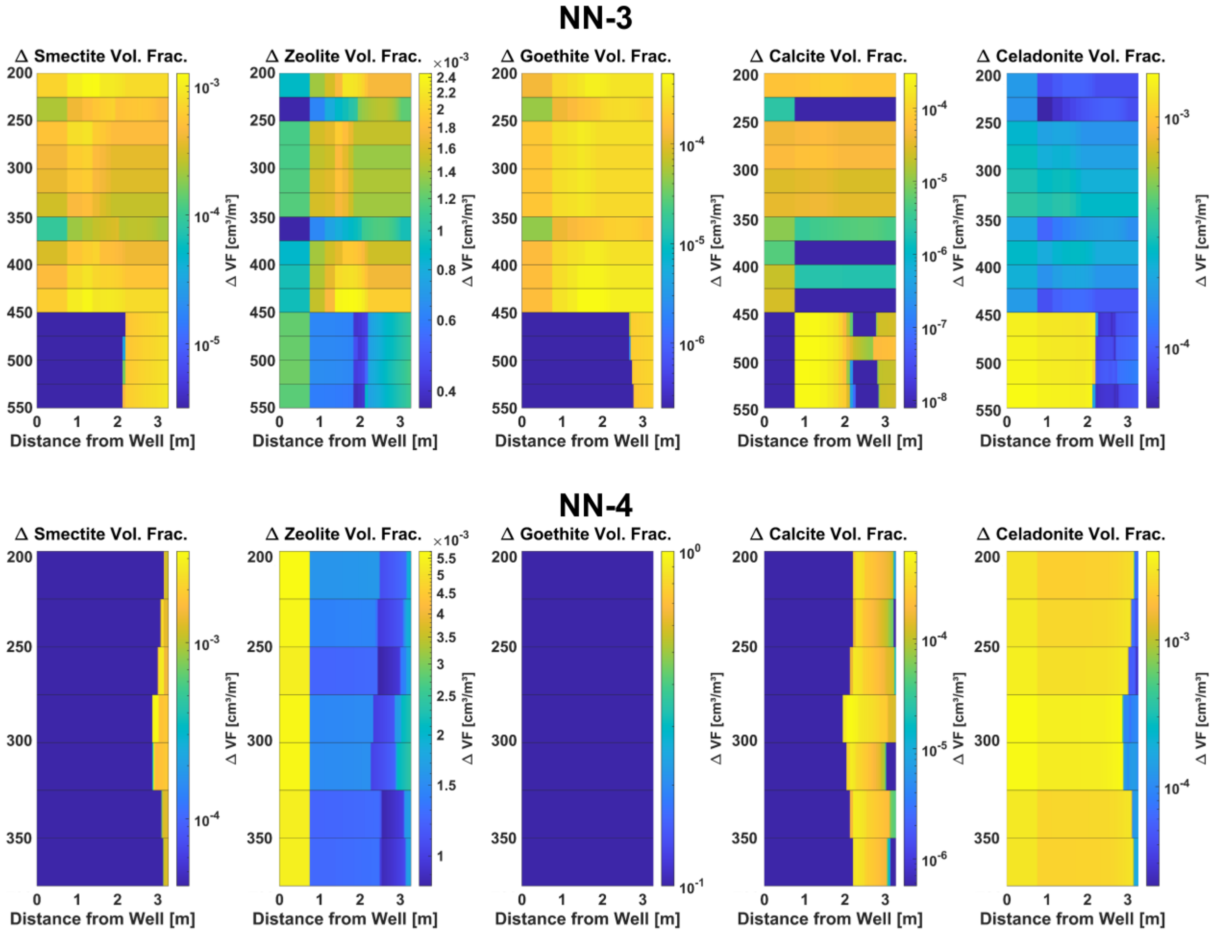

Figure S2: Magnitude and distribution of volume fraction change in secondary minerals of the NN-3 (top) and NN-4 (bottom) flow models. The models are parameterized as described in the main text for the RTM model results shown in Figure 4.

Figure S3: Source of Sulfur Upon Fluid-Rock Interaction

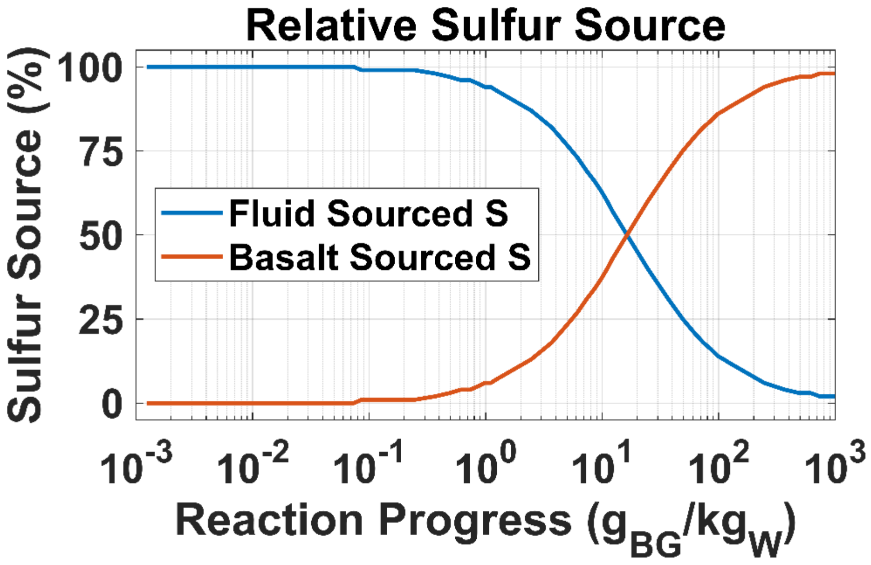

Figure S3: The percentage of S sourced from the H_2S charged injection fluid compared to the percentage of S sourced from the basaltic glass upon progressive alteration. At high water-rock ratios (i.e., low values along the x-axis), the injection fluid is the primary source of S. Upon alteration of 16 g of basaltic glass in 1 kg of water (0.13 mol_{BG}/kg_{W}), basalt sourced S becomes the dominant source of S. This modeling is done with NN-4 fluids and basaltic glass from 300-325 to establish an extreme case as this cell has the highest S mole ratio (0.00229 mol_{S}/mol_{BG}) of all cells in the reactive transport model.

Figure S4: Basaltic Glass Dissolution Rates

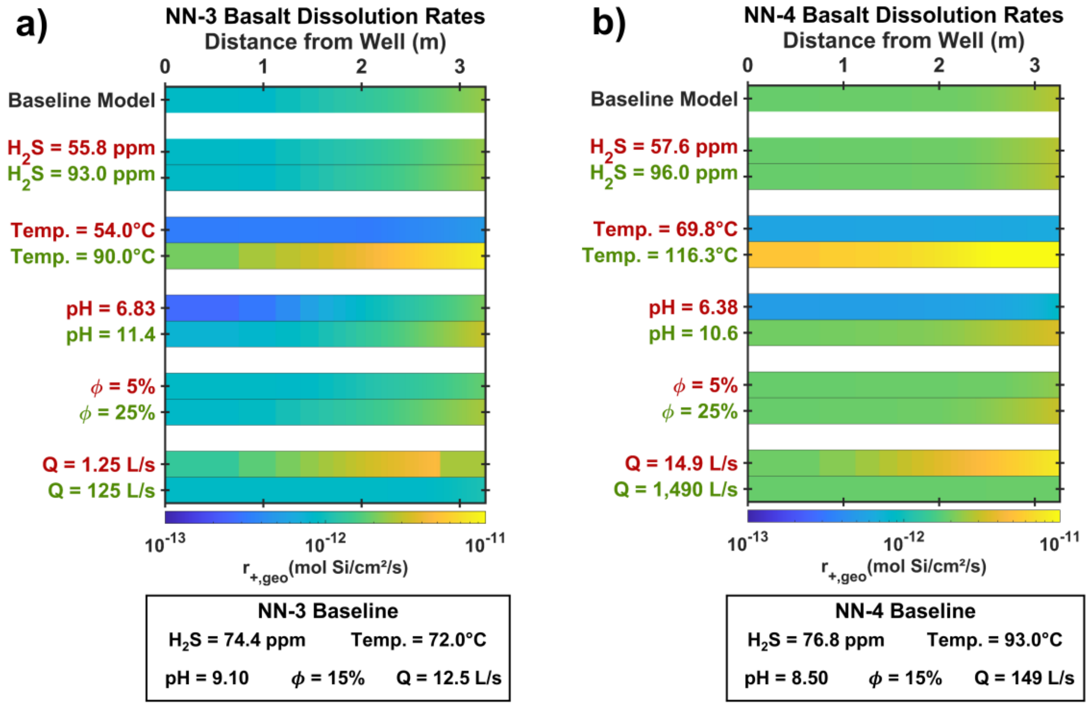

Figure S4: Compiled results of the surface area normalized basaltic glass dissolution rates for models of varying parameters in NN-3 and NN-4. The top row in both left and right figures is the basaltic glass dissolution rates given the measured fluid parameters (temperature, pH, H_2S concentration). The low parameter and high parameter estimates are -25% and +25% of the measured values, respectively, mid-estimate porosity, and the true injection rates with even allocation of the injection fluid to each model layer. All models are run with the average basalt composition over their respective well, NN-3 and NN-4.

Figure S5: Sulfide Formation Assuming Homogenous Porosity and Permeability

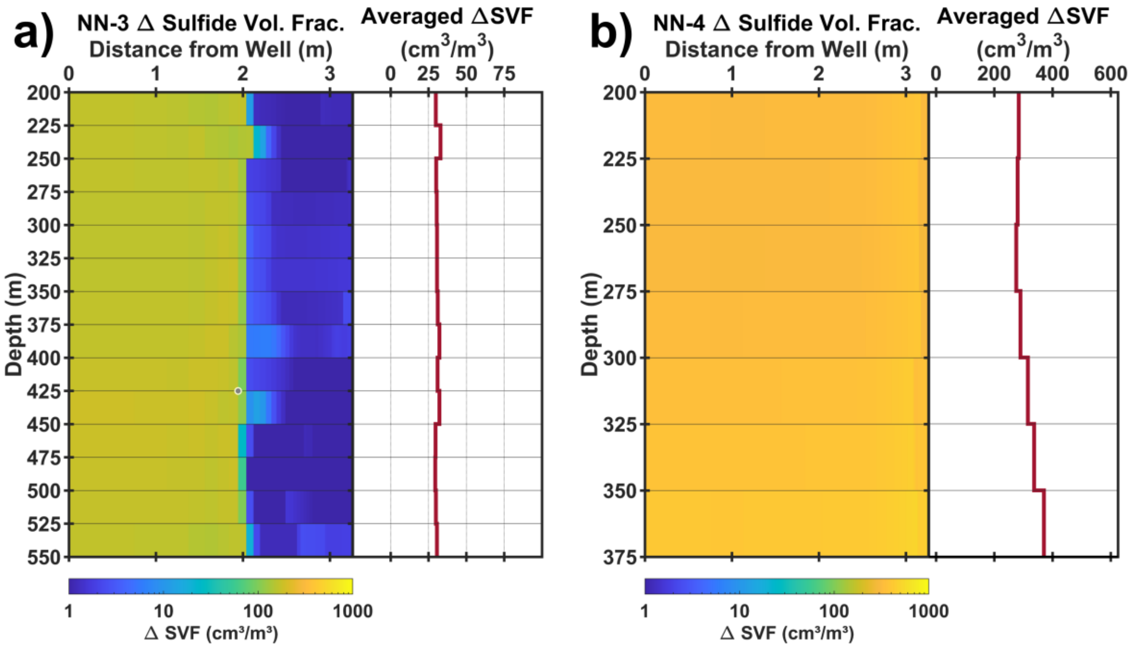

Figure S5: Change in sulfide volume fraction per cubic meter of basalt near the borehole after 40 days of injection in NN-3 (a) and NN-4 (b). The left panels show the distribution of change in sulfide volume fraction (SVF) as fluid flows laterally away from the injection wells. The right panels display the change in SVF at each depth as a weighted average over the flow path as described in Text S5. The models assume a homogeneous porosity of 15% and uniform volumetric flow allocation to each RTM layer, relative to each well's injection rate. The measured injection fluid compositions in wells NN-3 and NN-4 are used for each respective well mode (Table S4). The basaltic glass composition is defined from the measured whole rock compositions and are detailed in Table S3.

Figure S6: Sulfur Mineralization Efficiency

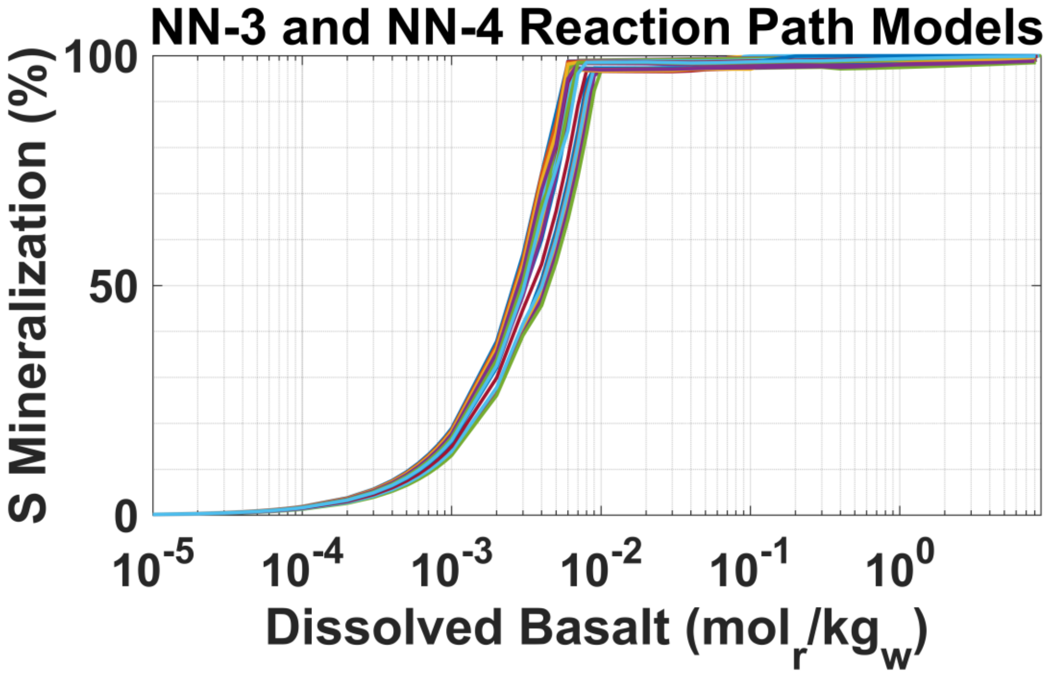

Figure S6: Reaction path models allowing secondary minerals (as listed in Table S5) to mineralize to equilibrium upon progressive basalt alteration in 1 kg of injection fluid. The percentage of S mineralized is calculated using the precipitated pyrite, pyrrhotite, and total S in the solution after each reaction step. A model is constructed for each of the basalt compositions measured in NN-3 and NN-4 to evaluate how basalt composition impacts S mineralization efficiency.

Figure S7: Comparison of Advective Transport to Advective-Dispersive Transport

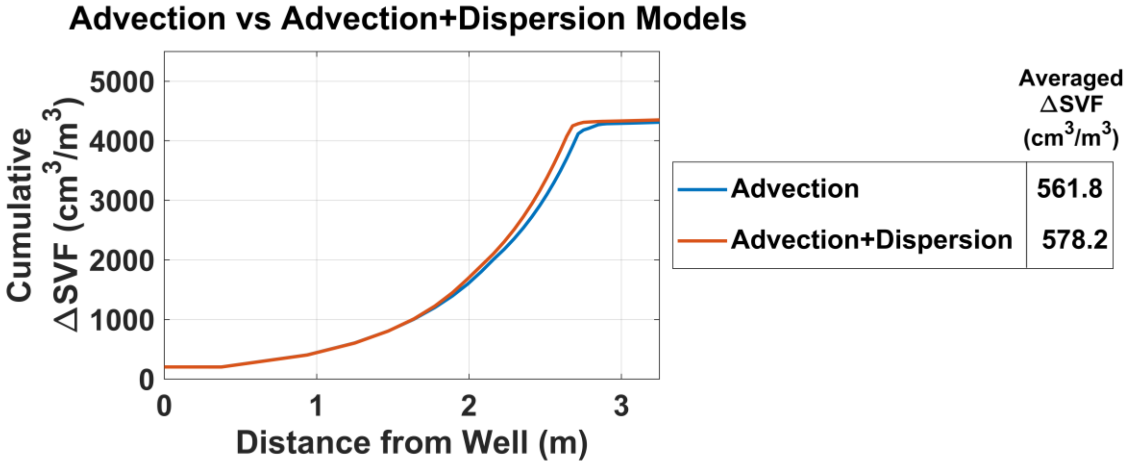

Figure S7: Change in the cumulative sulfide volume fraction for RTMs of NN-3, 525-550 m depth with advective transport compared to advective-dispersive transport with diffusion. Dispersity in each cell was set as 10% of the total flow distance (Appelo & Postma, 2004; Přikryl et al., 2018), and the diffusion coefficient is 0.3×10^{-9} m^2/s, the default value in PHREEQC (Parkhurst & Appelo, 2013).

Figure S8: Kinetic vs Equilibrium Precipitation

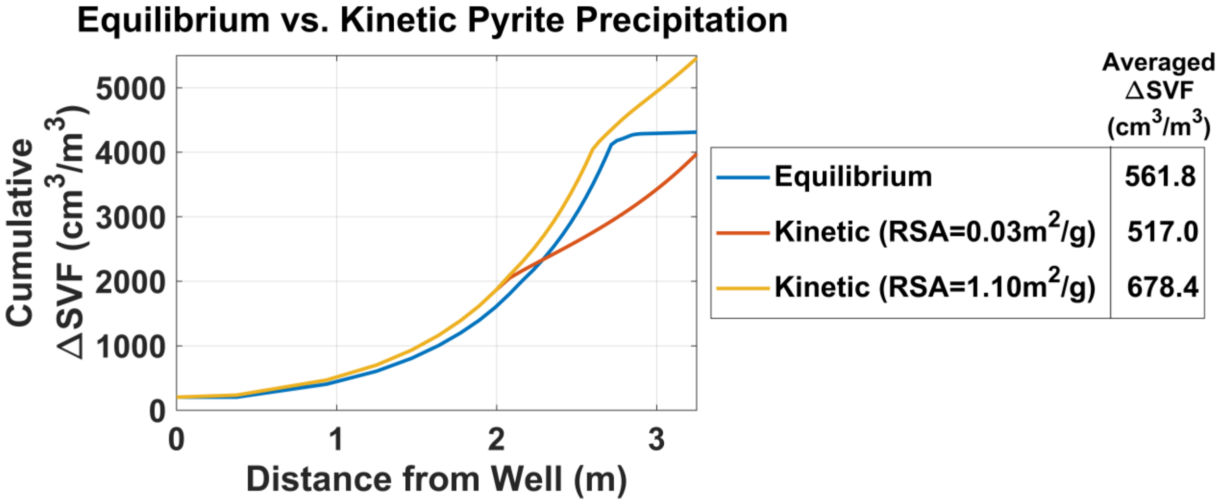

Figure S8: Cumulative change in sulfide volume fraction (SVF) for models where pyrite precipitation is either equilibrium driven (blue line), or kinetically controlled (yellow and orange lines). The kinetic rate constant for pyrite is 1.88×10^{-18} mol/m^2/s (Marty et al., 2010). The pyrite reactive surface areas represent the highest (1.10 m^2/g) and lowest (0.03 m^2/g) values compiled from a literature search of measured pyrite reactive surface areas presented in Beckingham et al. (2016). By using the extremes values for the range of pyrite reactive surface area, we test the sensitivity of H_2S mineralization to the reactive surface area and evaluate the assumption of equilibrium precipitation. Pyrite is the only sulfide allowed to mineralize. The results are shown for the reactive transport model of NN-3, 525-550 m depth.

Figure S9: Wireline Response Changes upon H_2S Injection

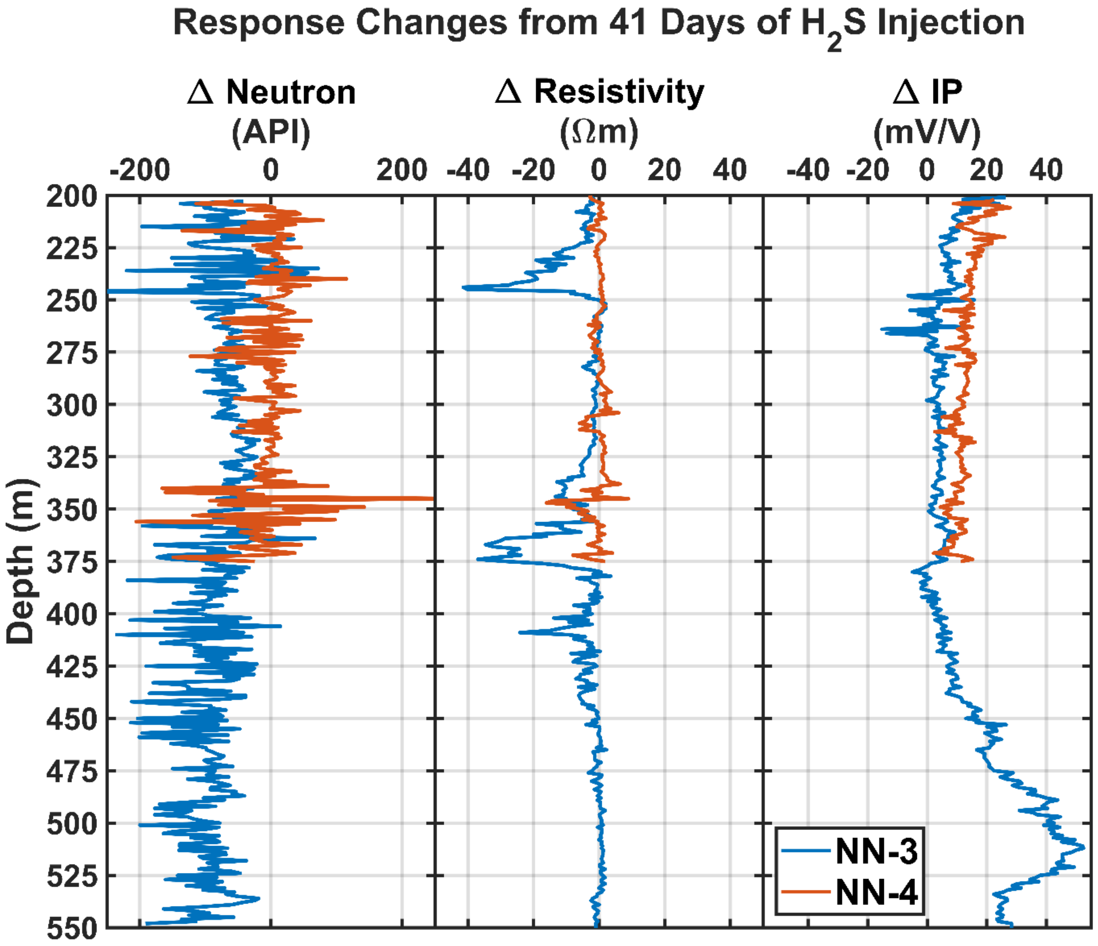

Figure S9: Wireline logging response changes after 40 days of H_2S injection at NN-3 and NN-4.

References (Supporting Information):

Appelo, C. A. J., & Postma, D. (2004). Geochemistry, groundwater and pollution, second edition. In *Geochemistry, Groundwater and Pollution, Second Edition*. https://doi.org/10.1201/9781439833544

Arnórsson, S., Bjarnason, J. Ö., Giroud, N., Gunnarsson, I., & Stefánsson, A. (2006). Sampling and analysis of geothermal fluids. *Geofluids*, *6*(3). https://doi.org/10.1111/j.1468-8123.2006.00147.x

Beckingham, L. E., Mitnick, E. H., Steefel, C. I., Zhang, S., Voltolini, M., Swift, A. M., Yang, L., Cole, D. R., Sheets, J. M., Ajo-Franklin, J. B., DePaolo, D. J., Mito, S., & Xue, Z. (2016). Evaluation of mineral reactive surface area estimates for prediction of reactivity of a multi-mineral sediment. *Geochimica et Cosmochimica Acta*, *188*. https://doi.org/10.1016/j.gca.2016.05.040

Galeczka, I. M., Stefánsson, A., Kleine, B. I., Gunnarsson-Robin, J., Snæbjörnsdóttir, S. Ó., Sigfússon, B., Gunnarsdóttir, S. H., Weisenberger, T. B., & Oelkers, E. H. (2022). A pre-injection assessment of CO2 and H2S mineralization reactions at the Nesjavellir (Iceland) geothermal storage site. *International Journal of Greenhouse Gas Control*, *115*. https://doi.org/10.1016/j.ijggc.2022.103610

Gómez-Díaz, E., Scott, S., Ratouis, T., & Newson, J. (2022). Numerical modeling of reinjection and tracer transport in a shallow aquifer, Nesjavellir Geothermal System, Iceland. *Geothermal Energy*, *10*(7). https://doi.org/10.1186/s40517-022-00217-3

Gysi, A. P., & Stefánsson, A. (2012). CO 2-water-basalt interaction. Low temperature experiments and implications for CO 2 sequestration into basalts. *Geochimica et Cosmochimica Acta*, *81*. https://doi.org/10.1016/j.gca.2011.12.012

Hafstað, Þ. H. (2003). Niðurrennslisholurnar NN-3 og NN-4 á Nesjavöllum. *ISOR: ÞHH-03-06*.

Hafstað, Þ. H., Stefánsson, H. Ö., & Franzson, H. (2015). Niðurrennsli á Nesjavöllum: Holur NN-6, NN-7 og NN-9. *ÍSOR-15035*.

Jeffery, G. H., Bassett, J., Mendham J, & Denney, R. C. J. (1989). *Vogel's textbook of quantitative chemical analysis* (5th ed.). Longman Scientific and Technical.

Jochum, K. P., Weis, U., Schwager, B., Stoll, B., Wilson, S. A., Haug, G. H., Andreae, M. O., & Enzweiler, J. (2016). Reference Values Following ISO Guidelines for Frequently Requested Rock Reference Materials. *Geostandards and Geoanalytical Research*, *40*(3). https://doi.org/10.1111/j.1751-908X.2015.00392.x

Kipp, K. L. (1987). HST3D—A computer code for simulation of heat and solute transport in three-dimensional ground-water flow systems. *U.S. Geological Survey Water-Resources Investigations Report 86–4095*.

Marty, N. C. M., Fritz, B., Clément, A., & Michau, N. (2010). Modelling the long term alteration of the engineered bentonite barrier in an underground radioactive waste repository. *Applied Clay Science*, *47*(1–2). https://doi.org/10.1016/j.clay.2008.10.002

Parkhurst, D. L., & Appelo, C. A. J. (2013). Description of input and examples for PHREEQC Version 3 — A computer program for speciation, batch-reaction, one-dimensional transport, and inverse geochemical calculations. In *U.S. Geological Survey Techniques and Methods, book 6, chapter A43*.

Přikryl, J., Marieni, C., Guðbrandsson, S., Aradóttir, E. S., Gunnarsson, I., & Stefánsson, A. (2018). H2S sequestration process and sustainability in geothermal systems. *Geothermics*, *71*. https://doi.org/10.1016/j.geothermics.2017.09.010

Roy, A., & Dhar, R. L. (1971). Radius of investigation in DC resistivity well logging. *Geophysics*, *36*(4). https://doi.org/10.1190/1.1440210

Stefánsson, A., Gunnarsson, I., & Giroud, N. (2007). New methods for the direct determination of dissolved inorganic, organic and total carbon in natural waters by Reagent-Free™ Ion Chromatography and inductively coupled plasma atomic emission spectrometry. *Analytica Chimica Acta*, *582*(1). https://doi.org/10.1016/j.aca.2006.09.001
